# Genome-wide association studies and QTL mapping for traits deviating from normal distribution

**DOI:** 10.1093/nsr/nwag184

**Published:** 2026-03-23

**Authors:** You Tang, Mingliang Li, Defu Liu, Liping Jiang, Qi Li, Jiabo Wang, Helong Yu, Shizhong Xu

**Affiliations:** Sanjiang Laboratory, Changchun 130000, China; Electrical and Information Engineering College, Jilin Agricultural Science and Technology University, Jilin 132000, China; Yazhouwan National Laboratory, Sanya 572024, China; College of Animal Science and Technology, Huazhong Agricultural University, Wuhan 430070, China; College of Information Technology, Jilin Agricultural University, Changchun 130000, China; Electrical and Information Engineering College, Jilin Agricultural Science and Technology University, Jilin 132000, China; Electrical and Information Engineering College, Jilin Agricultural Science and Technology University, Jilin 132000, China; Key Laboratory of Qinghai-Tibetan Plateau Animal Genetic Resource Reservation and Utilization, Sichuan Province and Ministry of Education, Southwest Minzu University, Chengdu, Sichuan 610000, China; College of Information Technology, Jilin Agricultural University, Changchun 130000, China; Department of Botany and Plant Sciences, University of California, Riverside, CA 92521, USA

**Keywords:** generalized linear mixed model, genome-wide association studies, non-normal trait, pseudo response variable, QTL mapping

## Abstract

Quantitative traits are the targets for genome-wide association studies (GWAS) and quantitative trait locus (QTL) mapping. After adjusting for systematic effects, these traits are assumed to be normally distributed so that typical linear models and linear mixed models (LMMs) can be used to detect markers associated with QTLs. Many traits in crops, animals and humans, however, do not follow the assumed normal distribution and many of them are not even continuously distributed. We developed statistical models and software packages to map QTLs and perform association studies for such non-normal traits under the generalized linear mixed model (GLMM) framework. We developed a pseudo response (PSR) method to estimate the polygenic variance by generating a pseudo-response variable that is treated as a conventional quantitative trait. We then scanned the genome for markers associated with the PSR variable in the usual LMM. The new method is called the pseudo response generalized linear mixed model (PSR-GLMM). We illustrated the method with the purple color trait of rice (binary trait) and a set of simulated non-normal traits. We then applied the method to four datasets: a binary trait from an *Arabidopsis* population, a binomial trait from a pig population, a Poisson trait from the same pig population and an ordinal trait from a dog population. A software package has been developed in R to perform GWAS and QTL mapping for binary, binomial, Poisson, and ordinal traits (PSR-GLMM/R), including normally distributed traits as a special case. The R package can also be applied to perform GLMM analysis for a general purpose beyond QTL mapping and GWAS.

## INTRODUCTION

Genome-wide association studies (GWAS) and quantitative trait locus (QTL) mapping are fundamental tools for gene discovery in plants, especially in agricultural crops. Associated markers detected by the tools provide target regions for further studies such as gene cloning and editing [1]. The basic statistical models for GWAS and QTL mapping are the linear models [2] and linear mixed models (LMMs) [3,4]. The linear models are often used by interval mapping (IM) [5] and composite interval mapping (CIM) [6] for linkage studies and by individual marker association studies as in PLINK [2]. The state-of-the-art models for GWAS are the LMM, which takes one step further to include correlated polygenic effects in the model as random effects and uses a marker inferred ‘kinship matrix’ to capture the random effects [7–10]. The same polygenic model has also been applied to QTL mapping in place of IM and CIM [11]. Therefore, the polygenic model has become the universal model for both linkage and association studies. The original linear mixed model [3,4] laid the foundation of the modern GWAS. The problem with this model is the high computational cost due to repeatedly inverting the covariance matrix of size $n \times n$ where *n* is the sample size.

In the span of two decades after the first mixed model GWAS [3,4], numerous advanced technologies have been developed to improve the computational efficiency, including EMMA and GEMMA that incorporate eigen decomposition of the kinship matrix into LMM [7,8,10], factored spectrally transformed linear mixed models (FaST-LMM) [12], efficient Bayesian linear mixed model (BOLT-LMM) [13], and the MLM-based tool for GWAS (fastGWA) [14]. These advanced algorithms can handle data at the UK Biobank scale (half million samples and millions of SNPs).

Theory and methods of GWAS are already very mature for typical quantitative traits. Many traits in plants, however, are binary, ordinal, or nominal. The advanced technology presented above are not appropriate for these discrete traits. Although some authors found that analyzing binary traits using IM, CIM, and LMM (methods typically used for quantitative traits) was not subject to noticeable loss in power and decrease in precision [15], others discovered that when the binary traits have a skewed distribution, the conventional LMM may not control the type I error properly [16,17]. Simulation studies also show that treating a binary or ordinal trait as a continuous variable will generate biased results, especially when the categories are not symmetric [18]. Therefore, generalized linear models (GLM) such as logistic regressions have been used to map QTL for binary traits. Typical examples include the BTL procedure in Statistical Analysis System (SAS) [19] and the logistic regression function in PLINK [2]. To properly control the type I error, the generalized linear mixed model (GLMM) should be the choice. The GLMM association test (GMMAT) for binary traits is one example [16]. The logistic ridge regression [20] is another example, where the ridge parameter was found in grid search *via* leave-one-out-cross-validation. Binary trait GWAS has been applied to pigs under the GLMM framework [21] using a software named GenABEL [22]. The most recent study of binary traits was the fast GWAS under the GLMM framework (fastGWA-GLMM) applied to the UK Biobank data (1/2 million individuals, 12 million variants) [23].

Binary traits are the most common discrete traits, but other discrete traits also appear in plants and animals. Some count data may be described by a Poisson distribution, for example, the number of lesions in a sampled leaf caused by some plant pathogen. Feather pecking behavior in chicken has been modeled as a Poisson trait for GWAS [24]. Some traits measured by ratio, for example, seed germination rate, can be modeled as a binomial trait. These additional traits have not been paid as much attention as the binary traits by the GWAS community. This project will develop the theory, the methods and the software package for all traits from the exponential family under the GLMM framework [25,26].

Multinomial traits (multiple categories) also belong to the exponential family and thus can be modeled *via* GLMM in linkage and association studies. There are two types of category traits: ordinal and nominal. Ordinal traits have at least three ordered categories, for example, severity of disease infection (unaffected, mild infection, severe infection and fatal). Nominal traits also have at least three categories, but different categories (levels) have no particular order, for example, leaf shape of a particular plant species (acicular, falcate, orbicular, and rhomboid). Linkage and association studies for ordinal traits have been reported [27,28]. The proportional odds logistic mixed model (POLMM) is specialized for ordinal trait association studies with polygenic background control [29]. Similar method and software package are also available, for example, the Julia OrdinalGWAS.jl. package [30]. In animals and humans, ordinal traits have been modeled with the random model *via* the marker IBD (identical by descent) matrix to test the marker variance and the additive relationship matrix (from pedigrees) to estimate the polygenic variance [31]. Wang *et al.* [32] modeled the ordinal phenotype and the ordinal genotype jointly to study the association. The most recent model for ordinal trait association studies is the collapsing and kernel methods in pedigree-structured samples [33]. These ordinal trait studies are based on the ordinal regression, the proportional odds, and the proportional hazards models [34].

The SAS is the world largest software organization for statistical analysis and financial management [35]. The cores of many statistical methods related to GWAS and QTL mapping can be found from SAS in the forms of SAS procedures. Each procedure performs a specialized task such as analysis of variances (ANOVA) and regression analysis (REG). A SAS procedure can deliver the results in the forms of datasets, tables, and graphics. As a result, a SAS procedure is like an R package, far beyond an R function. The MIXED procedure in SAS, with some modification, can perform QTL mapping and GWAS for quantitative traits with polygenic background control [11]. The GLIMMIX procedure, after significant technical improvement, can perform QTL mapping and GWAS for traits that deviate from normal distribution. The MIXED procedure handles all data analyses for continuous response variables (traits). The GLIMMIX procedure was designed to analyze data with discrete response variables (traits), in addition to continuous variables. However, QTL mapping and GWAS require repeated calls of the procedures for each genetic variant (marker) across the genome. Advanced levels of additional coding of the SAS procedures are required before the procedures can be applied to GWAS. A large proportion of the genetics community are non-SAS users. Instead of convincing them to use SAS, we developed an R package, which is open source that allows users to add new features of their own to the software. Although there are numerous R packages available for GWAS and QTL mapping [1], packages with the ability to analyze all non-normal traits within the same platform are rare. The new R package proposed in this project performs GWAS and QTL mapping for all types of traits, including normally distributed traits as special cases. Results of our R package have been validated with the high-quality SAS procedure PROC GLIMMIX.

## RESULTS

### Test the new model with a known gene in rice

**Figure 1.**
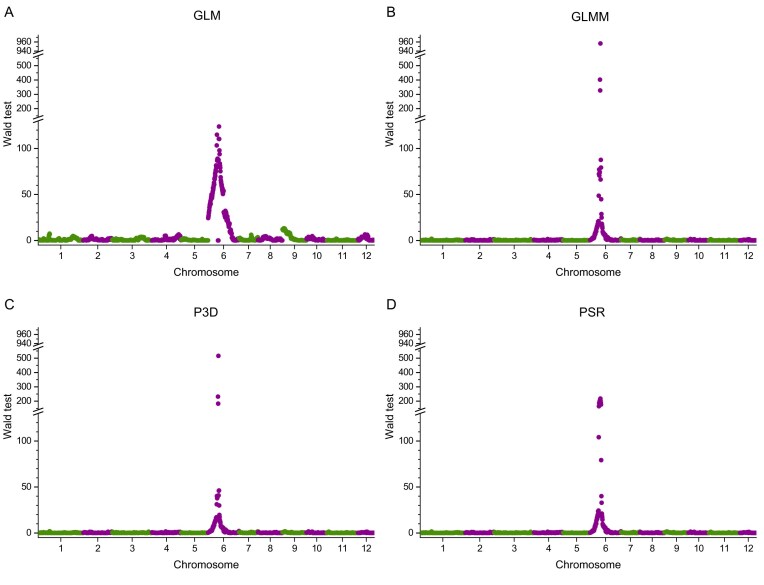
Manhattan plots of the purple color (binary) trait of a rice population consisting of 210 recombinant inbred lines. Four methods: (A) GLM; (B) GLMM; (C) P3D; and (D) PSR. The critical value of the Wald test for the genome of 1619 markers is 17.36.

The rice population consists of 210 recombinant inbred lines [36]. The trait is a binary trait controlled by a single mutant gene called *OsC1*. Plants with this gene develop purple leaves and stems (coded as 1) while plants lacking this gene show the wildtype green color (coded as 0). The binary phenotypes of the purple color trait of the 210 lines are presented in (Data S1). This gene is located on chromosome 6 and overlaps with a marker named bin868. The genotype file contains 1619 bins of the 210 lines (Data S2). The kinship matrix was calculated from all the 1619 bins (Data S3). Data analysis was conducted with the R package named PSR-GLMM/R developed in this study. Four methods were used to analyze the data: (A) the simple generalized linear model (GLM) without polygenic control; (B) the generalized linear mixed model (GLMM) with polygenic control, which is exact without any approximation; (C) population parameters previously determined (P3D), an approximate method; (D) the pseudo response (PSR) method, which is the method developed in this project. Method (A) is not comparable to the other three methods because the method lacks polygenic control. Figure 1 shows the Manhattan plots of the Wald test statistics. The GLMM method has a peak at the true location of the purple gene with the Wald test statistic of 956 and a *P*-value <1.0E-10. The two approximate methods (PSR and P3D) also show peaks at the true location with the Wald test statistics of 200 for PSR and 500 for P3D, both having a *P*-value <1.0E-10. Interestingly, the GLM method (without polygenic background control) missed the true gene (bin868) but picked bin871 (a few markers away from the true gene) with the Wald test statistic of 123. The true gene is embedded in bin868 and thus bin868 co-segregates with the gene. This makes the second derivative of the log- likelihood function with respect to the parameters ill-defined, leading to an extremely large estimation error of the model effect. As a result, the Wald test statistic became close to zero. The two neighboring bins are bin867 and bin869, which also have small Wald test statistics and large *P*-values. The computational efficiencies of the methods are very different. The exact method (GLMM) took 3301.3 s to scan the entire genome. The first approximate method (PSR) took 12.2 s, and the second approximate method (P3D) took 124.0 s to complete the genome scanning. The computational time of the GLM method is about the same as the PSR approximation.

### Test the new method with simulated data

**Figure 2.**
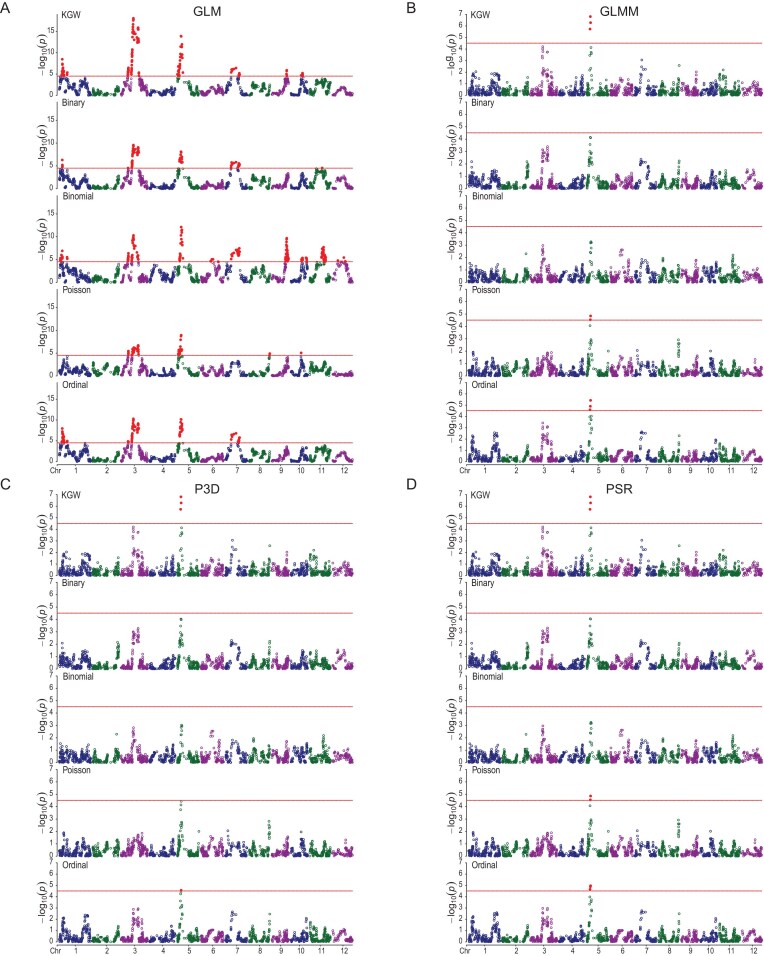
Manhattan plots of the KGW trait in a hybrid rice population and four discrete traits simulated from KGW. Panels A–D show results from GLM, GLMM, P3D, and PSR, respectively. Within each panel, the five stacked plots correspond to KGW, Binary, Binomial, Poisson, and Ordinal. The horizontal reference lines indicate the critical −log_10_(*p*) value of 4.5 for the genome of 1619 markers.

A hybrid population of rice [37] with $n = 278$ crosses from random pairing of 210 recombinant inbred lines was used to simulate different discrete traits for illustration of the new models. The phenotypes of four quantitative traits and five simulated discrete traits are given in Data S4, where the binomial trait occupies two columns (event and trial). The genotypes are shown in Data S5. The kinship matrix calculated from the genotype data is given in Data S6. We simulated four traits from the phenotypic values of the 1000 grain weight (KGW) trait (see method of simulation). We first mapped QTL for the KGW trait. Using the threshold of $- {\log }_{10}(0.05/1619) = 4.51$ for the $- {\log }_{10}(p)$ statistics or the threshold of ${\mathrm{qchisq}}( {1 - 0.05/1619,1} ) = 17.36267$ of the Wald test statistics, we identified one QTL in bin728 on chromosome 5 of the rice genome (Fig. 2). This QTL was previously reported and has been cloned [38]. Since all four simulated traits were based on the phenotypic values of KGW, the results of QTL mapping for the simulated traits should be similar or comparable to the result of the KGW trait. This is the purpose of the comparison.

**Figure 3.**
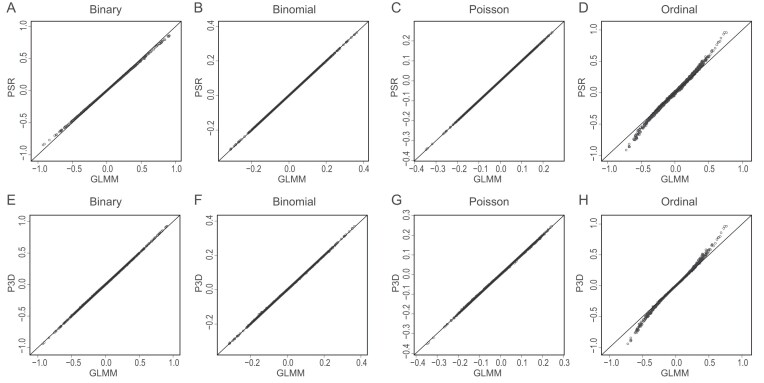
Comparisons of genome-wide marker effects between approximate methods and the exact GLMM for four discrete traits. (A–D) PSR vs. GLMM. (E–H) P3D vs. GLMM.

We compared the Manhattan plots of the four simulated traits with KGW for the GLM method (Fig. 2A), the GLMM method (Fig. 2B), the P3D method (Fig. 2C), and the PSR method (Fig. 2D). The Manhattan plots of the simulated traits have much the same patterns as that of the KGW trait. The GLM method used an entirely different model and thus the pattern of the plots are different from the three methods with polygenic background control. For the three methods (excluding GLM), the Poisson trait and the ordinal trait also show significance in the region around the KGW QTL on chromosome 5. The test statistics of the binary and binomial traits, however, did not reach the significance level, although both show obvious peaks in the QTL region. The GLM method also detected the QTL on chromosome 5, but many more were detected and many of them are false positives. The estimated marker effects of the entire genome from the PSR method are compared with the effects of the GLMM method for the four discrete traits (Fig. 3). Similar comparison of the P3D method with the GLMM method is shown in Fig. 3. All traits (except the ordinal trait) show consistency between the exact GLMM method and the approximate methods. Slight discrepancies are observed for the ordinal trait (Fig. 3D and H).

The QTL effects, the proportions of the phenotypic variance explained by the detected QTL, and other information in the QTL region are summarized in Table 1. For trait KGW, the true QTL is located in Bin728 whose Wald test statistic is 27.52 for the LMM method and 23.1664 for the P3D method. Since KGW is a quantitative trait, there is no PSR and thus the result for PSR is the same as the LMM method. The QTL heritability is 0.087 (see method), which is considered relatively high for a polygenic trait. For the simulated discrete traits, the peaks are around Bin728, some shifting a few markers from it. The estimated QTL heritability ranges from 0.03 to 0.06. Peaks of the binary and binomial traits did not reach the threshold of the test statistics but the peaks for the Poisson and ordinal traits are significant.

**Table 1.** Estimated QTL heritability and other information for the KGW trait and four discrete traits derived from KGW.

Trait	Method	QTL	Effect	StdErr	Wald	*P*-value	−Log_10_(*p*)	$\sigma _\xi ^2$	$\sigma _\varepsilon ^2$	MS_QTL_	MS_E_	$\sigma _{\rm QTL}^2$	$h_{\rm QTL}^2$
KGW	GLMM	Bin728	−1.0259	0.1955	27.5200	1.55E-07	6.8093	2.1662	0.5560	15.3035	0.5560	0.0532	0.0874
	PSR	Bin728	−1.0259	0.1955	27.5200	1.55E-07	6.8093	2.1662	0.5560	15.3035	0.5560	0.0532	0.0874
	P3D	Bin728	−1.0266	0.2133	23.1664	1.49E-06	5.8281	2.8200	0.5472	12.6766	0.5033	0.0439	0.0803
Binary	GLMM	Bin728	−0.9267	0.2333	15.7784	7.12E-05	4.1475	1.1951	0.0660	0.9420	0.0660	0.0024	0.0490
	PSR	Bin728	−0.8467	0.2160	15.3413	8.83E-05	4.0540	1.0575	0.0680	1.4503	0.0680	0.0052	0.0714
	P3D	Bin728	−0.9415	0.2409	15.2772	9.28E-05	4.0325	1.3348	0.0642	0.9038	0.0642	0.0033	0.0484
Binomial	GLMM	Bin731	−0.3156	0.0911	12.0074	0.00 053	3.2757	0.2363	0.1507	1.3618	0.1507	0.0049	0.0316
	PSR	Bin731	−0.3125	0.0905	11.9240	0.000 554	3.2562	0.2340	0.1509	1.4445	0.1509	0.0052	0.0334
	P3D	Bin731	−0.3158	0.0955	10.9408	0.000 941	3.0264	0.2831	0.1460	1.2217	0.1460	0.0044	0.0293
Poisson	GLMM	Bin729	−0.3469	0.0799	18.8338	1.43E-05	4.8447	0.1222	9.2129	150.8838	9.2129	0.5447	0.0558
	PSR	Bin729	−0.3453	0.0794	18.8960	1.38E-05	4.8601	0.1218	9.3877	145.0182	9.3877	0.5235	0.0528
	P3D	Bin729	−0.3528	0.0855	17.0242	3.69E-05	4.4330	0.1658	8.8948	132.0642	8.8948	0.4768	0.0509
Ordinal	GLMM	Bin729	0.7651	0.1655	21.3832	3.76E-06	5.4247	0.5821	0.8010	21.8209	0.8010	0.0379	0.0452
	PSR	Bin729	0.9560	0.2171	19.3860	1.07E-05	4.9706	1.4865	0.5212	19.3860	0.5212	0.0341	0.0613
	P3D	Bin729	0.9650	0.2299	17.6164	2.70E-05	4.5686	1.9151	0.4958	17.3063	0.4958	0.0303	0.0577

Table 2 compares the computational times among the three methods for the KGW trait and the four discrete traits. All experiments were conducted on an Ubuntu server powered by four Intel^®^ Xeon^®^ Gold 6138 processors (20 physical cores per processor at 2.00 GHz, with hyper-threading, yielding a total of 160 logical CPUs), 440GB of RAM, and an 8 TB hard disk. The GLMM method is computationally very costly for the discrete traits. The ordinal trait is even more costly because each individual has $C - 1$ linear predictors and thus $C - 1$ pseudo response variables. This is much like a multivariate analysis and the computational time gets increased as the number of categories increases. The P3D method has a significantly reduced computational time compared with the GLMM method. However, it is still not as efficient as the PSR method, which is the most efficient method. The general conclusion from the simulated data analysis is that both PSR and P3D are reasonably close to the GLMM method but with significantly shorter computational time, especially the PSR method, which is the primary focus of this project.

**Table 2.** Computational time comparisons between the approximate methods and the GLMM methods for the KGW trait of the hybrid rice and four simulated discrete traits derived from KGW.

Trait	GLMM (second)	PSR (second)	P3D (second)
KGW	4.8	–	–
Binary	6472.5	23.5	253.6
Binomial	2916.9	21.3	156.4
Poisson	3664.7	22.8	780.4
Ordinal	70 021.3	250.0	3600.0

### Power analysis

**Figure 4.**
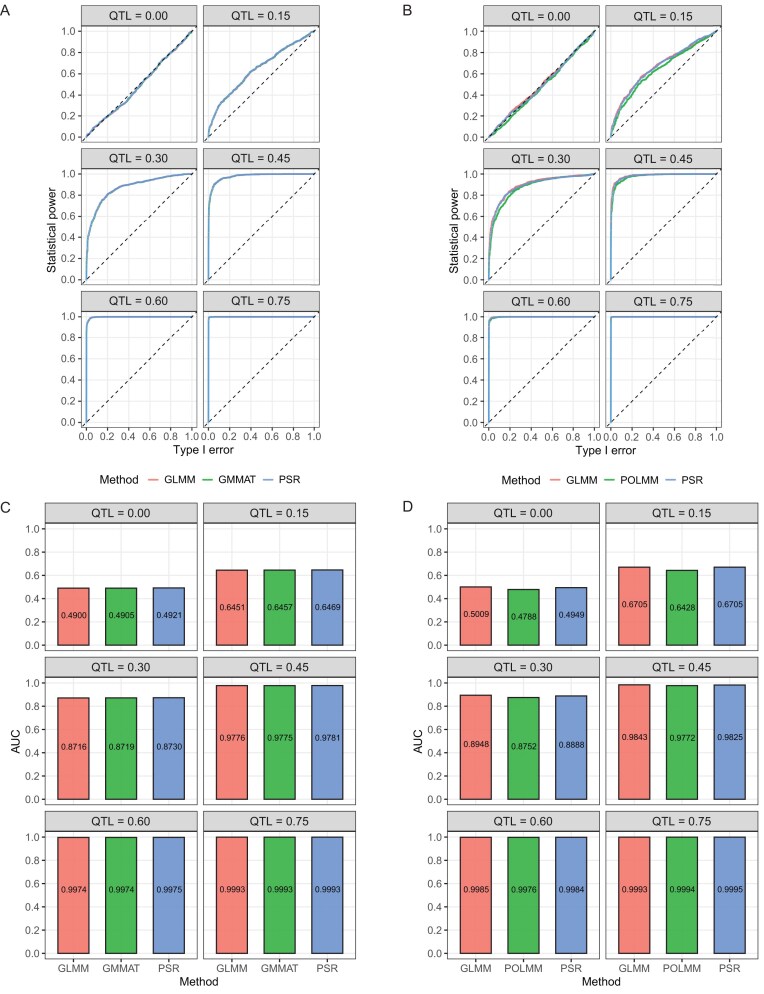
ROC curves and AUC comparison of three methods for binary (A and C) and ordinal (B and D) traits under six QTL effect sizes from 1000 replicated simulations.

Although the proposed new method (PSR) did not show noticeable difference from the exact method (GLMM) for the discrete traits simulated based on the KGW trait of the rice population, whether or not there is any loss in statistical power is still unknown. We performed a replicated simulation study particularly addressing the powers of different methods. We simulated a binary trait under various levels of the QTL effects and analyzed the trait using three methods: GLMM, PSR, and GMMAT [39]. We also simulated an ordinal trait and analyzed the data using three methods: GLMM, PSR, and POLMM [29]. Under each scenario the simulation was replicated 1000 times (see Method). The receiver operating characteristic (ROC) curves and areas under the curves (AUC) are shown in Fig. 4. The larger the area, the higher the power. Figure 4A shows the ROC curves for the binary trait and Fig. 4B shows the ROC curves of the ordinal trait under 6 levels of the QTL size, where GLMM and PSR are the two methods developed in this project and GMMAT was developed by Chen *et al.* [39] and POLMM was developed by Bi [29]. The corresponding AUCs are presented by Fig. 4C for the binary trait and Fig. 4D for the ordinal trait. All methods compared are equally powerful except that the POLMM method for ordinal trait analysis is slightly less powerful compared with other methods. The conclusion is that the PSR approximation does not lose any power compared with GLMM and other methods.

### Applications

We retrieved four datasets from three populations of plants and animals. (1) Binary trait: The binary trait is the Presence of *AvrRpm1* in *Arabidopsis thaliana. AvrRpm1* is the *Pseudomonas syringae* effector protein that activates the *Arabidopsis* intracellular innate immune receptor protein resistance to *Pseudomonas maculicola* 1. The phenotype was coded as 1 for presence and 0 for absence of *AvrRpm1*. The data were retrieved from AtPolyDB (call method 75, Horton *et al.* easyGWAS Public Data (mpg.de). The original data contained 1307 individuals and 214 051 markers (https://easygwas.ethz.ch/down/dataset/download/1/) [40]. After a strengthened quality control, we obtained 80 individuals and 216 063 SNP markers for data analysis. (2) Binomial trait: The binomial trait is the ratio of healthy piglets to the total piglets per litter of sows from a private swine company in Northeast China. We retrieved 200 sows (female pigs) for their first litter records and the DNA sequences of the sows with 57 612 SNP markers. The phenotype data were recorded as two variables, the healthy piglets (event) and total number of piglets (trial) per litter. (3) Poisson trait: The Poisson data were from the same pig population as the binomial trait. The phenotype is the number of stillbirths per litter. (4) Ordinal trait: The ordinal data were from a domestic dog population retrieved from a public database (https://doi.org/10.5061/dryad.266k4). The phenotype was the length of fur coded as 1 for short, 2 for median, and 3 for long. The sample size was 104 (individuals) and the genomic data consist of 159 592 SNP genotypes.

**Figure 5.**
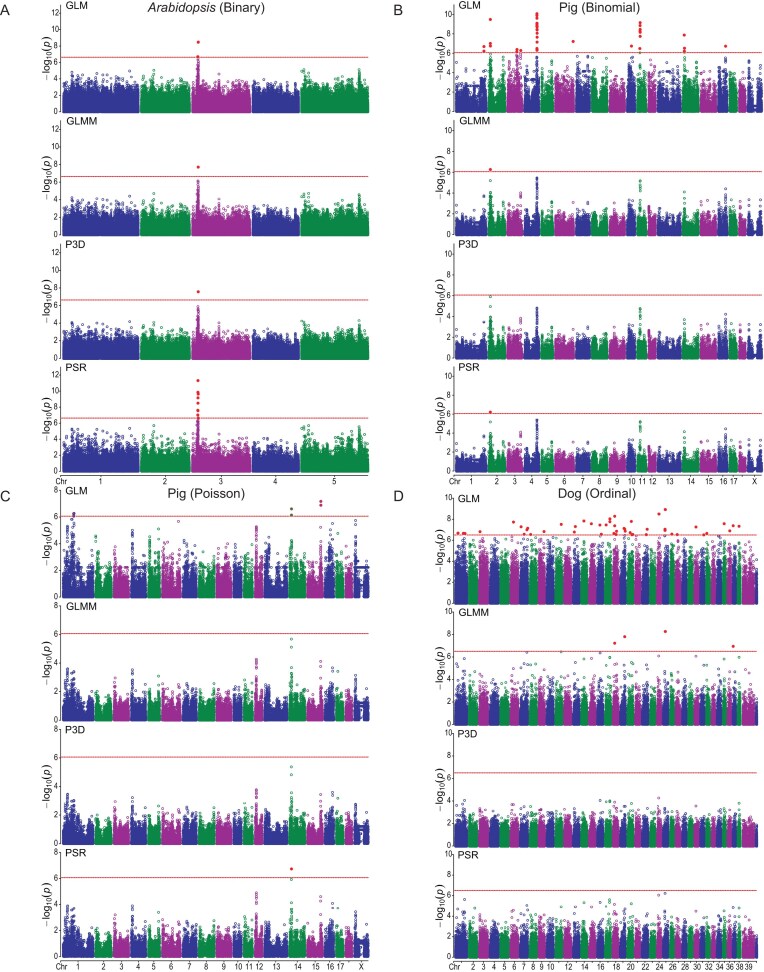
Manhattan plots of four discrete traits analyzed by four GWAS methods. Panels A–D show *Arabidopsis* (Binary), Pig (Binomial), Pig (Poisson), and Dog (Ordinal), respectively. Within each panel, the four stacked plots correspond to GLM, GLMM, P3D, and PSR. The horizontal dashed lines indicate the critical values of the test statistics.

The results of GWAS for the four traits are illustrated in Fig. 5 from four methods. Information on the detected QTL are listed in Table 3. For the binary trait, one marker from chromosome 3 was detected after Bonferroni correction by all four methods (Fig. 5A). This marker explains $h_{\rm QTL}^2 = 0.3597$ of the variance of the pseudo response variable for the GLMM method, $h_{\rm QTL}^2 = 0.5953$ of the pseudo response variance for the PSR method, and $h_{\rm QTL}^2 = 0.3443$ of the pseudo response variance for the P3D method. For the binomial trait, one marker was detected on chromosome 2 by three methods (GLM, GLMM, and PSR) and missed by P3D (Fig. 5B). The marker contributes $h_{\rm QTL}^2 = 0.0768$ of the variance for the pseudo response variable by GLMM, $h_{\rm QTL}^2 = 0.1006$ of the pseudo response by PSR and $h_{\rm QTL}^2 = 0.0772$ of the pseudo response variance by P3D. For the Poisson trait, one marker on chromosome 24 was detected only by GLM and PSR (Fig. 5C). The one detected by PSR contributes $h_{\rm QTL}^2 = 0.0645$ of the pseudo response variance (GLMM), $h_{\rm QTL}^2 = 0.1396$ of the pseudo response variance (PSR) and $h_{\rm QTL}^2 = 0.0646$ of the pseudo response variance (P3D). For the ordinal trait, the GLM method detected many markers, but only four of them were detected by GLMM and none of them were detected by the P3D and PSR methods (Fig. 5D). The four markers contribute to the pseudo variable variance by $h_{\rm QTL}^2 = 0.1422$, $h_{\rm QTL}^2 = 0.1335$, $h_{\rm QTL}^2 = 0.1587$, and $h_{\rm QTL}^2 = 0.1595$. We noticed that if a marker is detected by one of the three methods (GLMM, PSR, and P3D), the peaks often show similar patterns from the other methods that failed the detection. Overall, the conclusions from the four traits are quite consistent. Table 4 lists the computational times for the four traits from the four methods.

**Table 3.** Estimated QTL heritability and other information for four traits.

Trait	Method	QTL	Effect	StdErr	Wald	*P*-value	−Log_10_(*p*)	$\sigma _\xi ^2$	$\sigma _\varepsilon ^2$	MS_QTL_	MS_E_	$\sigma _{\rm QTL}^2$	$h_{\rm QTL}^2$
Binary	GLMM	85 167	1.1287	0.2010	31.5321	1.96E-8	7.7074	0.8445	0.0505	2.2409	0.0505	0.0284	0.3597
	PSR	84 948	−1.1681	0.1691	47.7350	4.88E-12	11.3117	0	0.0546	6.3393	0.0546	0.0802	0.5953
	P3D	85 167	1.1426	0.2055	30.9007	2.72E-08	7.5661	1.0525	0.0497	2.0613	0.0497	0.0261	0.3443
Binomial	GLMM	6 246	0.4388	0.0876	25.0910	5.47E-07	6.2621	0.7450	0.0440	0.7285	0.0440	0.0037	0.0768
	PSR	6 246	0.4030	0.0808	24.8762	6.11E-07	6.2137	0.6901	0.0447	0.9944	0.0447	0.0050	0.1006
	P3D	6 246	0.4457	0.0920	23.4658	1.27E-06	5.8957	0.9619	0.0401	0.6671	0.0401	0.0034	0.0772
Poisson	GLMM	42 994	1.4076	0.2961	22.6024	1.99E-06	5.7006	4.1499	0.3182	4.3687	0.3182	0.0220	0.0645
	PSR	42 994	1.2451	0.2388	27.1771	1.86E-07	6.7313	2.9608	0.3229	10.4406	0.3229	0.0525	0.1398
	P3D	42 994	1.4392	0.3131	21.1238	4.31E-06	5.3660	5.4316	0.2908	3.9962	0.2908	0.0201	0.0646
Ordinal	GLMM	89 963	−0.9065	0.1672	29.3836	5.94E-08	7.2264	0.2948	0.9297	32.6765	0.9297	0.1541	0.1422
	GLMM	95 942	−1.1893	0.2103	31.9723	1.56E-08	7.8058	0.3102	1.1414	37.3525	1.1414	0.1758	0.1335
	GLMM	117 534	−1.1308	0.1938	34.0297	5.43E-09	8.2654	0.2611	0.9543	38.0338	0.9543	0.1800	0.1587
	GLMM	150 968	−1.0060	0.1896	28.1451	1.13E-07	6.9487	0.3183	0.7950	31.8774	0.7950	0.1509	0.1595
	PSR	89 963	−0.8982	0.1997	20.2373	6.84E-06	5.1649	1.1721	0.4136	20.2372	0.4136	0.0962	0.1887
	PSR	95 942	−1.0160	0.2531	16.1107	5.97E-05	4.2237	1.3773	0.4064	16.1106	0.4064	0.0762	0.1579
	PSR	117 534	−1.1448	0.2291	24.9785	5.80E-07	6.2368	1.1191	0.3982	24.9785	0.3982	0.1193	0.2306
	PSR	150 968	−0.9774	0.2255	18.7847	1.46E-05	4.8347	1.2676	0.4075	18.7847	0.4075	0.0892	0.1796
	P3D	89 963	−0.8666	0.2259	14.7156	0.000 125	3.9031	2.2907	0.3287	14.9330	0.3287	0.0709	0.1774
	P3D	95 942	−1.0058	0.2820	0	1	0	2.2907	0.3370	12.7443	0.3370	0.0602	0.1516
	P3D	117 534	−1.1221	0.2602	0	1	0	2.2907	0.3108	18.5905	0.3108	0.0887	0.2221
	P3D	150 968	−0.9448	0.2536	0	1	0	2.2907	0.3299	13.9765	0.3299	0.0662	0.1672

**Table 4.** Computational time comparisons between the approximate methods and the GLMM methods for four traits of real data analysis.

Trait	GLMM (s)	PSR (s)	P3D (s)	GLM (s)
Binary	22 179.3	10 422.3	8150.2	666.9
Binomial	41 624.5	1976.5	4079.3	252.1
Poisson	99 468.4	1960.9	4906.6	399.3
Ordinal	893 317.7	1567.0	53 714.2	18 850.8

For the binary trait in the *Arabidopsis* population and the ordinal trait in the dog population, we also used the GMMAT [39] and POLMM [29] methods to scan the genomes. We also used the two methods analyzing the simulated binary and ordinal traits for comparison. The Manhattan plots are compared with our GLMM and PSR methods. The patterns of the Manhattan plots are very similar (Fig. S1).

## DISCUSSION

We developed an R package (PSR-GLMM/R) to implement the methods for association studies. The current program deals with binary, binomial, Poisson, and multinomial distributions along with normal distribution as a special case. The exponential family of distribution contains more than 30 different distributions [41], covering a wide range of traits. For example, chi-squared distribution, Wishart distribution, beta distribution, gamma distribution, geometric distribution, multinomial distribution, and so on. Some commonly observed agronomic traits may be handled more appropriately with a distribution other than normal. For example, the heading date trait of rice may be described with a gamma distribution, the number of lesion spots that causes a plant to die may be handled with a negative binomial distribution. With our PSR-GLMM/R, users do not need to call other programs for QTL mapping and GWAS because of the high versatility of the program.

While emphasizing the versatility of the new technology, we sacrifice the computational efficiency of the program. Some GWAS software packages are able to handle extremely large populations on the UK Biobank scale [2,12,13], but they are specialized to deal with some particular population structures and do not have the same versatility as this new technology. As the fast pace of improvement in computing power, especially in the anticipated quantum computing era, versatility will be more important than computability. One specialty of GWAS and QTL mapping is the genome scanning process which tests one marker at a time in an independent fashion. As a result, it is a perfect place to perform parallel computing, which can improve the computational speed hundred times, assuming 100 cores are activated the same time.

The primary focus of GWAS and QTL mapping is to detect associated markers. However, the size of a detected locus cannot be ignored. With increased population sizes, a small QTL, say explaining <1% of the phenotypic variance, may be detected. However, the biological significances of these tiny QTLs are really questionable. We need to focus on large QTL for follow up studies. The size of a QTL is measured by the QTL variance relative to the trait variance, that is, the QTL heritability. Two issues need to be clarified: (i) What is the correct method to estimate the QTL heritability? (ii) How do we take into consideration the bias associated with the significance test? For quantitative traits, the QTL heritability is defined as $h_{\rm QTL}^2 = \sigma _{\rm QTL}^2/(\sigma _{\rm QTL}^2 + \sigma _\varepsilon ^2)$. The estimated QTL heritability is $\hat{h}_{\rm QTL}^2 = \hat{\sigma }_{\rm QTL}^2/( {\hat{\sigma }_{\rm QTL}^2 + \hat{\sigma }_\varepsilon ^2} )$, where $\hat{\sigma }_{\rm QTL}^2$ is obtained from the ANOVA. For discrete traits like the binary one, the theoretical value of the residual variance is $\sigma _\varepsilon ^2 = 1$, and thus the QTL heritability is defined as $h_{\rm QTL}^2 = \sigma _{\rm QTL}^2/( {\sigma _{\rm QTL}^2 + 1} )$. However, $\hat{h}_{\rm QTL}^2 \not= \hat{\sigma }_{\rm QTL}^2/( {\hat{\sigma }_{\rm QTL}^2 + 1} )$, because the estimated residual variance is not exact unity but a value around unity. Therefore, the estimated QTL heritability for a discrete trait remains $\hat{h}_{\rm QTL}^2 = \hat{\sigma }_{\rm QTL}^2/( {\hat{\sigma }_{\rm QTL}^2 + \hat{\sigma }_\varepsilon ^2} )$, where both $\hat{\sigma }_{\rm QTL}^2$ and $\hat{\sigma }_\varepsilon ^2$ are obtained from the mean squares [42,43]. This QTL heritability is on the linear scale, not on the observed phenotype scale. Piepho [44] proposed a semivariance approach to estimate the R-squared in GLMM. That approach may be adopted to estimate the QTL heritability on the linear scale. There is a kind of bias associated with the significance test called the Beavis effect [45,46]. This bias should also be recognized when reporting the QTL heritability. Correction of the Beavis effect has been suggested [43,47]. Users are recommended to perform such correction for the reported loci.

The PSR method for GLMM is biased in terms of parameter estimation, especially for binary traits [48,49]. To show the level of bias and the effect of the bias on the GWAS result, we performed a simulation study (see Method for details of the simulation). The parameters are the polygenic variance (true value is 1.0) and the QTL effect (true value is 0.7 or 1.0, corresponding to $h_{\rm QTL}^2 = 0.10$ or $h_{\rm QTL}^2 = 0.18$). We compared the estimated parameters and the test statistics of four methods: (i) The pseudo response (PSR) method implemented with PROC GLIMMIX METHOD = RSPL in SAS; (ii) The Laplace (LAPLACE) method to approximate the marginal likelihood function implemented with PROC GLIMMIX METHOD = LAPLACE in SAS; (iii) A Bayesian generalized linear mixed model (BGLIMM) implemented with PROC BGLIMM in SAS under the default prior. (iv) Another Bayesian method implemented with the BGLR package in R [50] under the default prior. The BGLR package treated the underlying liability as missing values, which were then sampled from the conditional posterior distributions, which were truncated normal distribution [51]. The results are summarized in Fig. S2. The pseudo response method is indeed biased (downward) for the estimated QTL effect and the estimated polygenic variance. The Laplace method also shows slight biases for the estimated parameters, the two Bayesian methods (BGLIMM and BGLR) are unbiased. The *t*-test statistics (the two panels in the middle), however, are unbiased for all methods, including the pseudo response method. Therefore, the new PSR method is acceptable for detecting associated markers. The amount of bias appears to be proportional to the size of the QTL, the larger the QTL the higher the bias. In real data analysis, we do not expect to see many situations where the QTL heritability are >10%. Therefore, the small bias should be acceptable. The QUAD method in PROC GLIMMX cannot deal with the special covariance structure constructed from the kinship matrix. The Laplace method took about 22 min to complete the 100 simulations, while the pseudo response method took only 1.25 min for all the 100 simulations. The BGLIMM method took about 40 min to complete the 100 replicated simulations. The BGLR method took about 10 min to complete the analysis (much faster than PROC BGLIMM in SAS). Other than the PSR method, all other methods are too slow to perform GWAS for any realistic sample sizes with the usual marker density, say 20k markers. For example, the 100 replicated simulation experiment is equivalent to 100 markers. If we have 20 000 markers, we will need $200 \times 22 = 4400$ min (∼72 h) for the Laplace method. The BGLIMM will take $200 \times 40 = 8000$ min (∼133 h). The BGLR method will take $200 \times 10 = 2000$ min (∼33 h). The PSR-GLMM method will take about $200 \times 1.25 = 250$ min (∼4 h), is perhaps the only practical approach to performing GWAS for discrete traits. Since the *t*-test statistics are not biased for the PSR-GLMM method, we can use PSR-GLMM to scan the genome and detect the associated markers. A subsequent refined analysis may be conducted only for the detected markers with one of the unbiased methods. Alternatively, we may use the Laplace method under the null model to estimate the polygenic variance and generate the pseudo response variable. We then used the pseudo response variable in the QTL scanning stage.

Binary and ordinal traits can be analyzed via the Bayesian method by treating the liability as a missing quantitative trait whose value is sampled from a truncated normal posterior probability [51]. For the first time, we used the BGLR package in R [50] to evaluate the bias of QTL mapping for binary traits. Similar to the Bayesian method of Albert and Chib [51], BGLR also sampled the liability to from the truncated normal distribution. These Bayesian methods cannot be directly applied to other non-normal traits. Our PSR-GLMM method, however, is potentially extendable to analyze any traits in the exponential family. The current version of PSR-GLMM/R only deals with Poisson and binomial traits beyond the ordinal trait; it provides a platform where more non-normal traits can be added with ease in future versions.

Parallel to PSR-GLMM/R, we will develop a pipeline in SAS macro to repeatedly call the GLIMMIX procedure for QTL mapping and GWAS; such a pipeline is tentatively named PSR-GLMM/SAS. One advantage of PSR-GLMM/SAS will be the high versatility of handling over 30 different distributions. An obvious disadvantage of PSR-GLMM/SAS will be the slow computational speed relative to PSR-GLMM/R, although parallel computing can boost the speed. Readers can expect to see PSR-GLMM/SAS from this group of authors in the near future.

## METHODS

### Simulation studies

The 1000-grain weight (KGW) trait of a hybrid rice population (*n* = 278) was use to simulate a binary trait, a binomial trait, an ordinal trait and a Poisson trait. A total of 1619 SNP markers are available from the rice population. Since QTLs controlling KGW are already known, peaks of test statistics along the genome should appear around the known QTLs for the simulated non-normal traits. The results of the simulated non-normal traits serve as validations of the new methods.

### Generalized linear mixed models

Let *y* be a vector of phenotypic values of a non-normal trait that belongs to the exponential distribution family [41]. Define the expectation and variance of *y* by ${\mathrm{E}}( y ) = \mu $ and ${\mathop{\mathrm{var}}} (y) = \sum $, respectively. A link function connects μ with a linear predictor:


(1) \begin{eqnarray*}
\eta = {\zeta }^{ - 1}\left( \mu \right) = X\beta + Z\gamma ,
\end{eqnarray*}

where ${\zeta }^{ - 1}$ is the link function, $\eta = X\beta + Z\gamma $ is the linear predictor, *X* is a design matrix for fixed effects, $\beta $ is a vector of the fixed effects (including the intercept and the effect of a QTL under study), *Z* is a design matrix for random effects, and $\gamma $ is a vector of random effects with an assumed $N( {0,\sigma _\gamma ^2} )$ distribution.

### Linearization

The zeta function $\mu = \zeta ( \eta )$ can be approximated linearly via the Taylor series expansion at $\tilde{\eta } = X \tilde{\beta } + Z \tilde{\gamma }$ where $\tilde{\beta }$ and $\tilde{\gamma }$ are some starting values provided by the investigators [26]. Define $\Delta = \partial \mu /\partial \eta $ as the derivative of $\mu $ with respect to $\eta $ evaluated at $\eta = \tilde{\eta }$. We now define a pseudo response variable (PSR)


(2) \begin{eqnarray*}
\tilde{p} = {\tilde{\Delta }}^{ - 1}\left[ {y - \zeta \left( {\tilde{\eta }} \right)} \right] + \tilde{\eta }.
\end{eqnarray*}

The pseudo response variable is described with the following linear mixed model:


(3) \begin{eqnarray*}
\tilde{p} = X\beta + Z\gamma + \varepsilon .
\end{eqnarray*}

The residual variance is


(4) \begin{eqnarray*}
{\mathop{\mathrm{var}}} (\varepsilon ) &=& R = {\mathop{\mathrm{var}}} \left\{ {{{\tilde{\Delta }}}^{ - 1}\left[ {y - \zeta \left( {\tilde{\eta }} \right)} \right] + \tilde{\eta }} \right\}\\
&=& {\tilde{\Delta }}^{ - 1}\Sigma { \tilde{\Delta }}^{ - 1}
\end{eqnarray*}

which is only a function of $\tilde{\eta }$.

### Linear mixed model for pseudo response

We now treat Equation (3) as a typical linear mixed model. The expectation of $\tilde{p}$ is ${\mathrm{E}}( { \tilde{p}} ) = X\beta $ and the variance is


(5) \begin{eqnarray*}
{\mathop{\mathrm{var}}} (\tilde{p}) = V = ZG{Z}^T + R = Z{Z}^T \sigma _\gamma ^2 + R.
\end{eqnarray*}

After linearization, the conventional linear mixed model theory and method apply. We adopted the restricted maximum likelihood (REML) method for parameter estimation [52]. The restricted likelihood function for estimating the polygenic variance is


(6) \begin{eqnarray*}
{L}_R\left( {\sigma _\gamma ^2} \right) &=& - \frac{1}{2}\ln |V| { - \frac{1}{2}\ln |{X}^T{V}^{ - 1}X}
|\\
&&- \frac{1}{2}{( \tilde{p} - X \hat{\beta })}^T{V}^{ - 1}\left( { \tilde{p} - X\hat{\beta }} \right),
\end{eqnarray*}

where $\hat{\beta } = {({X}^T{V}^{ - 1}X)}^{ - 1}{X}^T{V}^{ - 1} \tilde{p}$, which are not parameters but functions of the polygenic variance. The linearization algorithm was originally provided by Wolfinger and O’Connell [26], who called the method the pseudo likelihood method. We call the method pseudo response (PSR) maximum likelihood instead.

### Doubly iterative algorithm for parameter estimation

**Figure 6.**
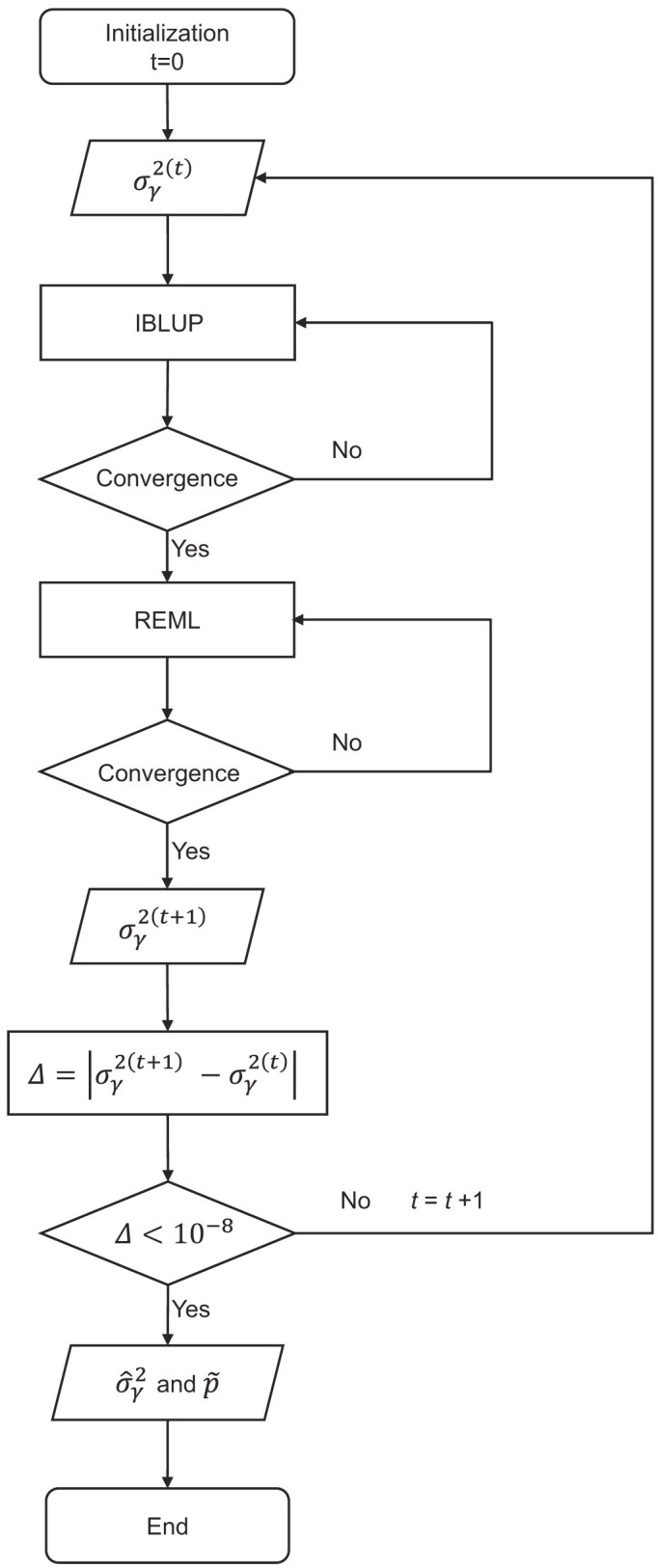
Flow chart of the doubly iterative algorithm of the generalized linear mixed model.

The linearization and generation of pseudo response variable converts the non-linear mixed model problem into a conventional linear mixed model problem. We called this linearization process iterative best linear unbiased prediction. However, this process depends on the polygenic variance. Given the pseudo response, we can run the conventional linear mixed model to estimate the variances using the REML method. Given the estimated variance, we then go back to the first loop to update the pseudo response variable. The iterative process continues until the parameters converge to a convergence criterion. The estimation procedure involves two inner loops and one outer loop and thus it is called the doubly iterative algorithm (see the flow chart in Fig. 6). We now describe this algorithm in detail.

Further methodological details are provided in Note S1.

## Supplementary Material

nwag184_Supplemental_Files

## SOFTWARE AVAILABILITY

The PSR-GLMM/R software platform, together with user manuals, demonstration code, full source code, example datasets, and bibliographic references, is freely accessible at http://106.13.144.206/psrglmm/index.html. PSR-GLMM/R implements generalized linear mixed-model (GLMM) procedures specifically optimized for genome-wide association studies (GWAS) of non-normally distributed traits. Users can click on these links to conveniently learn about algorithm principles, download program code, access example data for quick testing, and refer to relevant research literature for deeper understanding and application of the model. In the revised version of the R program, we added a general-purpose module that can be used for GLMM beyond QTL mapping and GWAS. Note S2 provides a short description on how to convert a linear predictor with a kinship matrix ($\eta = X\beta + \xi $) into a conventional linear predictor for generalized linear mixed model ($\eta = X\beta + Z\gamma $). The time complexities of all three methods (GLMM, PSR, and P3D) are presented in Note S3.
