# Supplementary material for: Genome-wide association studies and QTL mapping for traits deviating from normal distribution
Source: Natl Sci Rev. 2026 Mar 23;13(10):nwag184. doi: 10.1093/nsr/nwag184 (PMC13218386; doi:10.1093/nsr/nwag184)
Supplement: nwag184_Supplemental_Files [file nwag184_supplemental_files.zip › Supplementary Notes.docx]

**Genome-wide Association Studies and QTL Mapping for Traits Deviating from Normal Distribution**

You Tang, Mingliang Li, Defu Liu, Liping Jiang, Qi Li, Jiabo Wang, Helong Yu and Shizhong Xu

**Supplementary Note S1: Detailed Methods**

**S1.1 Design of the simulation experiments**

We used an existing hybrid rice dataset to test the new generalized linear mixed model for QTL mapping. The data consist of $n=278$ hybrid rice varieties evaluated in two years (1998 and 1999) for a trait called thousand grain weight (KGW) [1]. Genotypes of 1619 SNPs of the 278 hybrids were inferred from the genotypes of their 210 inbred parents. A kinship matrix was inferred from all 1619 SNP genotypes of the hybrids [2]. The additive effect of each SNP was estimated and tested with the Wald test statistics. Define *y* as the mean phenotypic values of the 278 hybrids measured in the two replications. The response variable *y* is a vector of length $n=278$. The phenotype data, the genotype data and the kinship matrix are given in **Supplementary Data S4**, **Data S5** and **Data S6**, respectively. In addition to the KGW trait, the experimenters also collected several other quantitative traits, including yield per plant (YIELD), grain number per plant (GRAIN), and tiller number per plant (TILLER). A major QTL for the KGW trait has been mapped to the rice genome, which is located in chromosome 5 [3]. We first performed the conventional QTL mapping procedure with polygenic background control [4] for KGW. The result for this trait was used as a control to validate the new methods of QTL mapping for four simulated discrete traits based on the phenotypes of KGW. From the phenotypic values of the KGW trait, we simulated the following discrete traits: binary, binomial, Poisson, and ordinal traits.

*Binary trait:* The average phenotypic value of the KGW trait is 25.06g. We assigned a binary phenotypic value to each individual according to the phenotype of KGW. The binary phenotype was assigned to 1 if KGW > 25.6 and 0 if KGW < 25.6, and the sequence of 0 and 1 forms the binary response variable.

*Binomial trait:* To simulate the binomial trait, we generated two random numbers per individual, one being the trial denoted by $t_{j}$ and the other being the event denoted by $e_{j}$. The trial was simulated from a Poisson distribution with expectation of $\lambda=5$, i.e., $t_{j}\sim\text{Poisson}\left( \lambda\right)$. The event was generated from $e_{j}\sim\text{Binomial}\left( t_{j},\mu_{j} \right)$, where $\mu_{j}$ is a proportion defined as a function of the KGW value. Let $y_{j}$ be the KGW value for individual *j*, then $\mu_{j}=(y_{j}-y_{min})/(y_{max}-y_{min})$. The binomial trait was defined as the ratio of the event to the trial. However, the input data points for the binomial trait are represented by the events and trials (two columns in **Supplementary Data S4**).

*Poisson trait*: The Poisson trait was simulated from the phenotypic value of KGW with mean (and the variance) defined by $\mu_{j}=5\times(y_{j}-y_{min})/(y_{max}-y_{min})$. where $y_{j}$ is the phenotypic value of KGW from the *j*th individual, i.e., $p_{j}\sim\text{Poisson}\left( \mu_{j} \right)$.

*Ordinal trait*: We partitioned the KGW trait phenotype into three categories with equal frequencies. The quantiles that partition the sample into three parts of equal size were 24.3465 and 25.899431. The ordinal data point was defined as $o_{j}=1$ if $y_{j}\leq24.35$, $o_{j}=2$ if $24.35<y_{j}\leq25.89$, and $y_{j}=3$ if $y_{j}>25.89$.

**S1.2 Generalized linear mixed models**

Let $y_{j}$ be the phenotypic value of a trait measured from the *j*th individual for $j=1,\cdots,n$ where $n$ is the sample size. Assume that $y_{j}$ belongs to the exponential distribution family [5]. Denote the expectation and variance of $y_{j}$ by $E\left( y_{j} \right)=\mu_{j}$ and $\mathrm{var} (y_{j})=\Sigma_{j}$, respectively. Define a linear predictor for individual *j* by

$\begin{aligned} \eta_{j}=X_{j}\beta+Z_{j}\gamma\#\left( 1 \right) \end{aligned}$

where $X_{j}$ is a set of independent variables capturing the fixed effects ($\beta$) and $Z_{j}$ is a set of independent variables capturing the random effects ($\gamma$). In QTL mapping and GWAS, the fixed effects are covariates such as the year effects, location effects, age effects and so on. The random effects are effects of genetic variants such as SNPs, InDels, structural variations (SV) and so on. The random effects are assumed to follow a multivariate normal distribution, i.e., $\gamma\sim N\left( 0,G \right)$, where $G=I\sigma_{\gamma}^{2}$ and $\sigma_{\gamma}^{2}$ is called the polygenic variance shared by all genetic variants. The connection between the expectation and the linear predictor is the key for analyzing non-normal traits with the generalized linear mixed model. This connection is called the zeta function whose inverse is called the link function as denoted by $\mu_{j}=\zeta\left( \eta_{j} \right)$ and $\eta_{j}=\zeta^{-1}\left( \mu_{j} \right)$, respectively, where $\zeta^{-1}$ is called the link function. The link function is merely symbolic, and the original zeta function is the one used in all derivations of GLMM. We now extend the above formulas to the entire population using matrix notations, $\mu=\zeta\left( \eta\right)$ and $\eta=\zeta^{-1}\left( \mu\right)$. For normally distributed quantitative traits, the relationship is $\mu=I\left( \eta\right)=X\beta+Z\gamma$ and thus the link function is simply the identity matrix.

**S1.3 Linearization**

The zeta functional $\zeta\left( \eta\right)$ can be approximated linearly via the Taylor series expansion of $\mu$ at $\eta=\tilde{\eta}$ for $\tilde{\eta}=X\tilde{\beta}+Z\tilde{\gamma}$ where $\tilde{\beta}$ and $\tilde{\gamma}$ are some starting values provided by the investigators [6]. The first order Taylor series expansion is

$\begin{aligned} \mu\approx\zeta\left( \tilde{\eta} \right)+\tilde{\Delta}\left( \eta-\tilde{\eta} \right)\#\left( 2 \right) \end{aligned}$

where $\tilde{\Delta}={\partial\mu}/{\partial\eta}$ is the derivative of $\mu$ with respect to $\eta$ evaluated at $\eta=\tilde{\eta}$. Rearrangement of terms in equation (2) leads to

$\begin{aligned} \tilde{\Delta}^{-1}\left[ \mu-\zeta\left( \tilde{\eta} \right) \right]+\tilde{\eta}=\eta\#\left( 3 \right) \end{aligned}$

We now replace $\mu$ in the left hand side of equation (3) by *y* and denote this by

$\begin{aligned} \tilde{p}=\tilde{\Delta}^{-1}\left[ y-\zeta\left( \tilde{\eta} \right) \right]+\tilde{\eta}\#\left( 4 \right) \end{aligned}$

Given $\eta=X\beta+Z\gamma$, we now have the following linear mixed model,

$\begin{aligned} \tilde{p}=X\beta+Z\gamma+\varepsilon\#\left( 5 \right) \end{aligned}$

where $\tilde{p}$ is called the pseudo response (PSR) variable. The residual variance is

$\begin{aligned} \mathrm{var} (\varepsilon)=R=\mathrm{var} \left\{ \tilde{\Delta}^{-1}\left[ y-\zeta\left( \tilde{\eta} \right) \right]+\tilde{\eta} \right\}=\tilde{\Delta}^{-1}\Sigma\tilde{\Delta}^{-1}\#(6) \end{aligned}$

which is only a function of $\tilde{\eta}$, not a function of $\eta$.

An alternative but very intuitive derivation of the linearization was provided by Finney [7]. He started with the residual on the binary scale, $y-\tilde{\mu}$, and then converted this residual into a residual on the linear scale, $\left( {\partial\eta}/{\partial\mu} \right)\left( y-\tilde{\mu} \right)$. This residual is added to the linear predictor, $\tilde{\eta}+\left( {\partial\eta}/{\partial\mu} \right)\left( y-\tilde{\mu} \right)$, which forms the pseudo response variable defined here. Since $\partial\eta/\partial\mu=(\partial\mu/\partial\eta)^{-1}=\tilde{\Delta}^{-1}$, the pseudo response variable is then written as $\tilde{p}=\tilde{\Delta}^{-1}\left( y-\tilde{\mu} \right)+\tilde{\eta}$, which is identical to equation (4). This intuitive derivation is much simpler and easier to understand than the one given by Wolfinger and O’Connell [6].

**S1.4 Linear mixed model for pseudo response**

We now treat equation (5) as a typical linear mixed model. The expectation of $\tilde{p}$ is $E\left( \tilde{p} \right)=X\beta$ and the variance is

$\begin{aligned} \mathrm{var} (\tilde{p})=V=ZGZ^{T}+R=ZZ^{T}\sigma_{\gamma}^{2}+R\#(7) \end{aligned}$

After linearization, the conventional linear mixed model theory and method apply. We adopted the restricted maximum likelihood (REML) method for parameter estimation [8]. The restricted likelihood function for estimating the polygenic variance is

$$\begin{aligned} L_{R}(\sigma_{\gamma}^{2})=-\frac{1}{2}\ln|V|-\frac{1}{2}\ln|X^{T}V^{-1}X|-\frac{1}{2}(\tilde{p}-X\hat{\beta})^{T}V^{-1}\left( \tilde{p}-X\hat{\beta} \right)\#(8) \end{aligned}$$

where $\hat{\beta}=(X^{T}V^{-1}X)^{-1}X^{T}V^{-1}\tilde{p}$, which are not parameters but functions of the polygenic variance. Any iterative algorithms can be used to solve the REML solution of the polygenic variance. The linearization algorithm was originally provided by Wolfinger and O’Connell [6], who called the method the pseudo likelihood method. We call the method pseudo response (PSR) maximum likelihood instead.

**S1.5 Doubly iterative algorithm for parameter estimation**

The linearization and generation of pseudo response variable converts the non-linear mixed model problem into a conventional linear mixed model problem. We called this linearization process iterative best linear unbiased prediction (IBLUP). However, this process depends on the polygenic variance. Given the pseudo response, we can run the conventional linear mixed model to estimate the variances using the restricted maximum likelihood (REML) method. Given the estimated variance, we then go back to the first loop to update the pseudo response variable. The iterative process continues until the parameters converge to a convergence criterion. The estimation procedure involves two inner loops and one outer loop and thus it is called the doubly iterative algorithm (see the flow chart in **Figure 6**). We now describe this algorithm in detail.

**S1.5.1 Iterative best linear unbiased prediction (IBLUP)**

Given the polygenic variance ($\sigma_{\gamma}^{2}$), we need to find the pseudo response variable ($\tilde{p}$), which depends on the current value of the linear predictor, $\tilde{\eta}=X\tilde{\beta}+Z\tilde{\gamma}$. We start with $\eta^{\left( k \right)}=X\beta^{\left( k \right)}+Z\gamma^{\left( k \right)}$ for $k=0$ to update the pseudo response,

$\begin{aligned} p^{\left( k \right)}=\frac{1}{\Delta^{\left( k \right)}}\left[ y-\zeta\left( \eta^{\left( k \right)} \right) \right]+\eta^{\left( k \right)}\#\left( 9 \right) \end{aligned}$

From $\tilde{p}=p^{\left( k \right)}$, we then update $\eta^{\left( k+1 \right)}=X\beta^{\left( k+1 \right)}+Z\gamma^{\left( k+1 \right)}$ using the BLUE of the fixed effects,

$\begin{aligned} \beta^{\left( k+1 \right)}=\left( X^{T}V^{-1}X \right)X^{T}V^{-1}\tilde{p}\#\left( 10 \right) \end{aligned}$

and the BLUP of the random effects,

$\begin{aligned} \gamma^{\left( k+1 \right)}=\sigma_{\gamma}^{2}Z^{T}V^{-1}\left( \tilde{p}-X\beta^{\left( k+1 \right)} \right)\#\left( 11 \right) \end{aligned}$

where *V* is defined in equation (7) and *R* is defined in equation (6). From the updated BLUE and BLUP, we then updated the linear predictor,

$\begin{aligned} \eta^{\left( k+1 \right)}=X\beta^{\left( k+1 \right)}+Z\gamma^{\left( k+1 \right)}\#\left( 12 \right) \end{aligned}$

which is used to update the pseudo response again using

$\begin{aligned} p^{\left( k+1 \right)}=\frac{1}{\Delta^{\left( k+1 \right)}}\left[ y-\zeta\left( \eta^{\left( k+1 \right)} \right) \right]-\eta^{\left( k+1 \right)}\#\left( 13 \right) \end{aligned}$

The process needs to iterate until $p^{\left( k+1 \right)}\approx p^{\left( k \right)}$. Upon convergence, we conclude the IBLUP loop.

**S1.5.2 Restricted maximum likelihood (REML) estimation of the polygenic variance**

The pseudo response variable ($\tilde{p}$) is treated as the “observed phenotype” that is used to estimate the polygenic variance component. The restricted log likelihood function is given in equation (8). Iterations are required to obtain the REML estimate of $\sigma_{\gamma}^{2}$ via the Newton method,

$\begin{aligned} \sigma_{\gamma}^{2\left( k+1 \right)}=\sigma_{\gamma}^{2\left( k \right)}-\left[ \frac{\partial^{2}L_{R}\left( \sigma_{\gamma}^{2} \right)}{\partial(\sigma_{\gamma}^{2})^{2}} \right]^{-1}\left[ \frac{\partial L_{R}\left( \sigma_{\gamma}^{2} \right)}{\partial\sigma_{\gamma}^{2}} \right]\#\left( 14 \right) \end{aligned}$

However, this REML estimate of $\sigma_{\gamma}^{2}$ depends on $\tilde{p}$. Therefore, we need the doubly iterative algorithm to complete the generalized linear mixed model analysis.

**Figure 6** is the flow chart of the doubly iterative algorithm for variance component estimation under the generalized linear mixed model. After the iteration converges, we have the estimated parameter ($\hat{\sigma}_{\gamma}^{2}$), the BLUE of the fixed effects and the BLUP of the random effects. In addition, we have the pseudo response variable $\tilde{p}=\left[ \begin{matrix} \tilde{p}_{1} & \cdots& \tilde{p}_{n} \end{matrix} \right]^{T}$ and the residual covariance matrix $R=\tilde{\Delta}^{-1}\Sigma\tilde{\Delta}^{-1}$. We are ready to introduce three approaches to scanning the whole genome for QTL.

**S1.6 Special distributions of non-normal traits**

The distributions of traits and the link functions determine the derivatives of the expectation with respect to the linear predictors, $\Delta={\partial\mu}/{\partial\eta}$, and the trait variances $\mathrm{var} (y)=\Sigma$. These quantities determine the pseudo response variable and the residual covariance structure. We now introduce $\Delta$ and $\Sigma$ for the following distributions and their link functions.

**S1.6.1 Binary trait**

The *j*th observation is denoted by $y_{j}=1$ for presence and $y_{j}=0$ for absence of a binary trait. The variance is $\mathrm{var} (y_{j})=\Sigma_{j}=\mu_{j}(1-\mu_{j})$. For probit link function, the expectation is $\mu_{j}=\Phi\left( \eta_{j} \right)$ and the derivative is $\Delta_{j}=\phi\left( \eta_{j} \right)$, where $\Phi\left( \right)$ and $\phi\left( \right)$ are the standardized normal distribution and density, respectively. For the logit link function, the expectation is $\mu_{j}=(1+e^{-\eta_{j}})^{-1}$ and the derivative is $\Delta_{j}=e^{-\eta_{j}}/(1-e^{-\eta_{j}})^{2}=\mu_{j}(1-\mu_{j})$. Occasionally, a student t distribution may be used for the expectation. The corresponding link function (inverse t distribution) is called the robit link function [9], which is more robust than the probit and logit link functions. When the degree of freedom is 7 or relatively large, the robit link function is equivalent to the logit or probit link function.

**S1.6.2 Binomial trait**

The phenotypic values are determined by two numbers, $m_{j}$ being the number of events and $n_{j}$ being the number of trials. The *j*th observation is denoted by $y_{j}={m_{j}}/{n_{j}}$. The expectation is $E\left( y_{j} \right)=\mu_{j}$and the variance is $\mathrm{var} (y_{j})=\Sigma_{j}=\mu_{j}(1-\mu_{j})/n_{j}$. Probit and logit link functions are often used for binomial traits. Again, a robit link function may also apply to binomial traits.

**S1.6.3 Poisson trait**

Traits measured by counts may be modeled as Poisson traits. The phenotypic value is denoted by $y_{j}=0,1,2,\cdots,\infty$. The expectation and variance are the same, $E(y_{j})=\mathrm{var} (y_{j})=\mu_{j}$. Under the log link function, $\mu_{j}=\exp(\eta_{j})$ and $\log(\mu_{j})=\eta_{j}$. The derivative of the expectation with respect to the linear predictor is $\Delta=\partial\mu_{j}/\partial\eta_{j}=\exp(\eta_{j})=\mu_{j}$.

**S1.6.4 Ordinal trait**

The ordinal variable is denoted by $S_{j}=1,\cdots,C$ for $j=1,\cdots,n$, where $n$ is the sample size and *C* is the number of ordered categories. Let $y_{j}=\left\{ y_{jk} \right\}$, $\forall k=1,\cdots,C$, be a $C\times1$ vector to indicate the phenotype of individual $j$. The *k*th element of $y_{j}$ is defined as

$y_{jk}=\left\{ \begin{matrix} 1 \\ 0 \end{matrix} \right.\begin{matrix} \\ \end{matrix}\begin{matrix} \text{for} \\ \text{for} \end{matrix} \begin{matrix} S_{j}=k \\ S_{j}\neq k \end{matrix}$

The expectation of $y_{jk}$ is defined as

$\mu_{jk}=E\left( y_{jk} \right)=\zeta\left( \alpha_{k}+X_{j}\beta+Z_{j}\gamma\right)-\zeta\left( \alpha_{k-1}+X_{j}\beta+Z_{j}\gamma\right)$

where $\alpha_{k}$ $\left( \alpha_{0}=-\infty\text{ and }\alpha_{C}=+\infty\right)$ is the *k*th intercept, where $\zeta\left( \right)$ is a cumulative distribution function (either a normal distribution or a logistic distribution) and $X_{j}\beta+Z_{j}\gamma$ is the linear predictor. The relationship between the expectation and the intercepts are sketched in **Supplementary Figure S3**. We often use $\eta_{jk}=\alpha_{k}+X_{j}\beta+Z_{j}\gamma$ to denote the linear predictor. Note that $\eta_{k}$ differs from $\eta_{k’}$ only by different thresholds, the fixed and random effects are the same across different categories. An ordinal trait with *C* categories requires $C-1$ linear predictors, which is in contrast to the non-normal traits described earlier. To demonstrate the specialty of ordinal traits, we now use an ordinal trait with $C=3$ as an example. Take the probit link function for example, the expectations of $y_{jk}$ are

$$\begin{aligned} \begin{matrix} \mu_{j1}=\Phi\left( \eta_{1} \right)-\Phi\left( \eta_{0} \right)=\Phi\left( \eta_{1} \right) \\ \mu_{j2}=\Phi\left( \eta_{2} \right)-\Phi\left( \eta_{1} \right)=\Phi\left( \eta_{2} \right)-\Phi\left( \eta_{1} \right) \\ \mu_{j3}=\Phi\left( \eta_{3} \right)-\Phi\left( \eta_{2} \right)=1-\Phi\left( \eta_{2} \right) \end{matrix}\#\left( 15 \right) \end{aligned}$$

due to the fact that $\alpha_{0}=-\infty$ and $\alpha_{3}=+\infty$. Let $\mu_{j}=E\left( y_{j} \right)=\left\{ \mu_{jk} \right\}$ be a $C\times1$ vector of the expectations of $y_{j}$. The variance matrix of $y_{j}$ is

$\begin{aligned} \mathrm{var} (y_{j})=\Sigma_{j}=\mathrm{diag}(\mu_{j})+\mu_{j}\mu_{j}^{T}\#\left( 16 \right) \end{aligned}$

The method to be developed requires the inverse of $\Sigma_{j}$. However, the inverse does not exist, and we must use a generalized inverse, one of which is

$\begin{aligned} \Sigma_{j}^{-}=\left[ \mathrm{diag}\left( \mu_{j} \right) \right]^{-1}=\mathrm{diag}\left( \mu_{j}^{-1} \right)\#\left( 17 \right) \end{aligned}$

Creating the pseudo response variables requires the partial derivatives of $\mu_{j}$ with respect to $\eta_{j}$, which is a $C\times\left( C-1 \right)$ matrix denoted by $\Delta_{j}$,

$\begin{aligned} \Delta_{j}=\left[ \begin{matrix} {\partial\mu_{j1}}/{\partial\eta_{j1}} & {\partial\mu_{j1}}/{\partial\eta_{j2}} \\ {\partial\mu_{j2}}/{\partial\eta_{j1}} & {\partial\mu_{j2}}/{\partial\eta_{j2}} \\ {\partial\mu_{j3}}/{\partial\eta_{j1}} & {\partial\mu_{j3}}/{\partial\eta_{j2}} \end{matrix} \right]=\left[ \begin{matrix} \phi\left( \eta_{j1} \right) & 0 \\ -\phi\left( \eta_{j1} \right) & \phi\left( \eta_{j2} \right) \\ 0 & -\phi\left( \eta_{j2} \right) \end{matrix} \right]\#\left( 18 \right) \end{aligned}$

We also need the inverse of $\Delta_{j}$ to build the pseudo response variables. However, $\Delta_{j}^{-1}$ does not exist. A similar situation has occurred in desired gain selection index (Yamada et al. 1975 and Itoh & Yamada 1986) where the genetic covariance matrix is not invertible. A generalization of the desired gain index to the pseudo response variable leads to

$\begin{aligned} \Delta_{j}^{-1}=(\Delta_{j}^{T}\Sigma_{j}^{-}\Delta_{j})^{-1}\Delta_{j}^{T}\Sigma_{j}^{-}\#\left( 19 \right) \end{aligned}$

Therefore, the pseudo response variable for ordinal data analysis is

$$\begin{aligned} \tilde{p}_{j}=\Delta_{j}^{-1}(y_{j}-\tilde{\mu}_{j})+\tilde{\eta}_{j}=(\Delta_{j}^{T}\Sigma_{j}^{-}\Delta_{j})^{-1}\Delta_{j}^{T}\Sigma_{j}^{-}(y_{j}-\tilde{\mu}_{j})+\tilde{\eta}_{j}\#\left( 20 \right) \end{aligned}$$

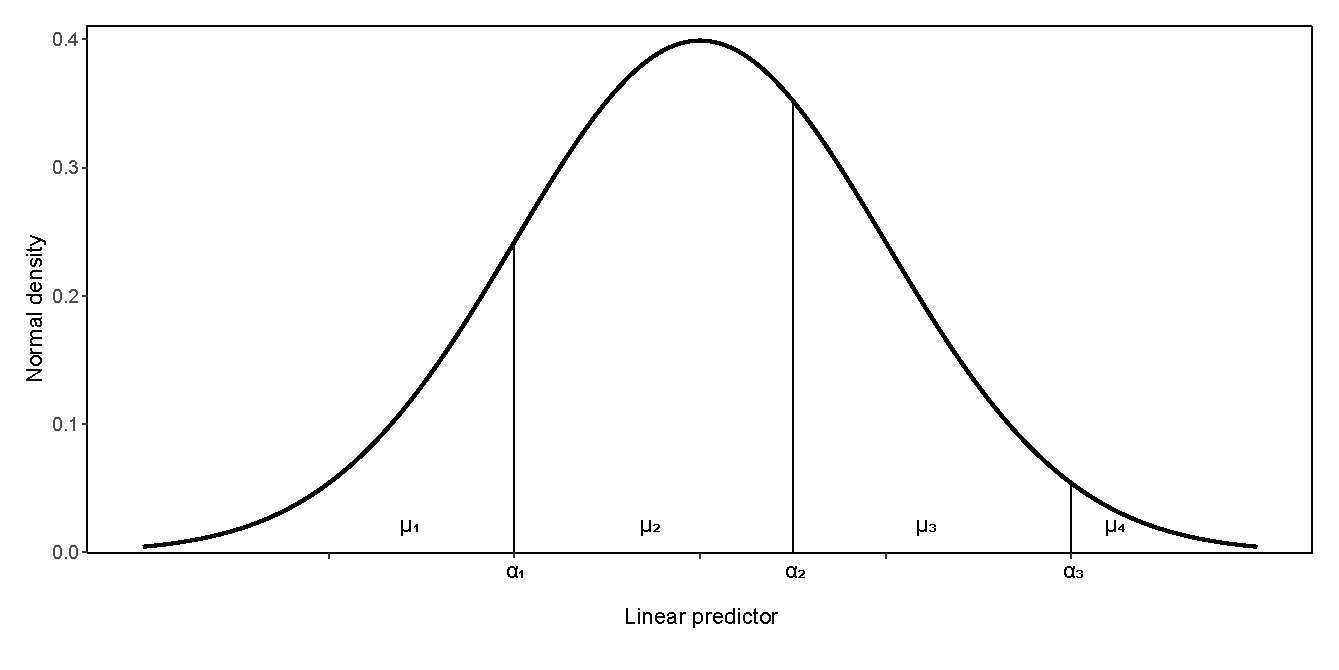


**Supplementary Figure S3** The threshold model for ordinal traits. The three thresholds cut the normal distribution into four categories. The area of each category represents the expectation of the ordinal trait for that category.

**S1.7 Scanning the genome for associated markers**

**S1.7.1 Generalized linear mixed model (GLMM)**

The doubly iterative algorithm described early applies to each marker so that the pseudo response variable is written as

$\begin{aligned} \tilde{p}=X\beta+Z_{k}\gamma_{k}+\xi+\varepsilon\#\left( 21 \right) \end{aligned}$

where $Z_{k}$ is the genotype indicator variable for marker *k* and $\gamma_{k}$ is the marker effect for $k=1,\cdots,m$ and *m* is the total number of markers. The marker effect is now treated as a fixed effect. The random polygenic effect is $\xi=Z\gamma$, where $Z$ is an $n\times m$ matrix of marker genotype indicators and $\gamma$ is an $m\times1$ effects of genome-wide markers. The variance of the polygenic effect is $\mathrm{var} (\xi)=ZZ^{T}\sigma_{\gamma}^{2}=K\sigma_{\xi}^{2}$, where $K=ZZ^{T}$ is the kinship matrix and $\sigma_{\xi}^{2}=\sigma_{\gamma}^{2}$ is just an alternative notation for the polygenic variance. Note that each marker has two effects in model (21), the standing alone one ($\gamma_{k}$) is a fixed effect and the one in $\xi=Z\gamma$ is a random effect. The expectation is $E\left( \tilde{p} \right)=X\beta+Z_{k}\gamma_{k}$ and the variance is $\mathrm{var} (\tilde{p})=V=K\sigma_{\xi}^{2}+R$. Let us define $P=V^{-1}-V^{-1}X\left( X^{T}V^{-1}X \right)X^{T}V^{-1}$. The estimated marker effect is $\hat{\gamma}_{k}=(Z^{T}PZ)^{-1}Z^{T}P\tilde{p}$ with a variance of $\mathrm{var} (\hat{\gamma}_{k})=(Z^{T}PZ)^{-1}$. Therefore, the Wald test statistic is $W_{k}=\hat{\gamma}_{k}^{2}/\mathrm{var} (\hat{\gamma}_{k})$. Every marker will go to this process (including generating $\tilde{p}$ and estimating the polygenic variance) and the doubly iterative algorithm is implemented for every single marker. No approximations are involved and thus this method is called the exact method labelled as GLMM. No fast algorithms are available to improve the efficiency of computation.

**S1.7.2 Pseudo response (PSR)**

Under the null model, i.e., $\tilde{p}=X\beta+\xi+\varepsilon$, we perform the doubly iterative algorithm to generate the pseudo response variable $\tilde{p}$ (an $n\times1$ vector), which is treated as “the observed normal response variable” or “phenotype vector”. We then fit this $\tilde{p}$ to the conventional linear mixed model (LMM) for every marker of the genome. By calling the LMM to scan the genome, we have avoided GLMM and thus tremendously reduced the computational time. The polygenic variance is constantly updated with a fixed $\tilde{p}$ when each marker is scanned. This approximation converts the GLMM into LMM in genome scanning. All special tricks applied to LMM can be applied here to improve the computational efficiency. The typical trick is the factored spectrally transformed linear mixed models (FaST-LMM), which performs eigen decomposition to the kinship matrix [10–12]. This trick, after some level of modification, is used here. Recall that the variance of model (21) is $V=K\sigma_{\xi}^{2}+R$, where $R$ is a diagonal matrix (binary, binomial and Poisson) or block diagonal matrix (ordinal and nominal). The FaST-LMM trick cannot be applied directly here because $R\neq I$. Let $K=UDU^{T}$ where $U$ is the eigenvector matrix and *D* is the eigenvalue matrix (diagonal). Pre-multiplying all terms in model (21) by $U^{T}$ leads to $U^{T}\tilde{p}$ whose variance is $\mathrm{var} (U^{T}\tilde{p})=V=D\sigma_{\xi}^{2}+U^{T}RU$. Although $D\sigma_{\xi}^{2}$ is diagonal, $U^{T}RU$ is not, and thus directly inverting *V* is unavoidable. Let us define $R^{-\frac{1}{2}}$ as the Cholesky decomposition of $R^{-1}$, i.e., $(R^{-\frac{1}{2}})^{T}R^{-\frac{1}{2}}=R^{-1}$. Define $K^{*}=(R^{-\frac{1}{2}})^{T}KR^{-\frac{1}{2}}$ and decompose $K^{*}$ so that $K^{*}=U^{*}D^{*}U^{*T}$. Now, let us define $L=R^{-\frac{1}{2}}U^{*T}$ and pre-multiply all terms in (21) by $L^{T}$, we get

$\begin{aligned} \tilde{p}^{*}=L^{T}\tilde{p}=L^{T}X\beta+L^{T}Z_{k}\gamma_{k}+L^{T}\xi+L^{T}\varepsilon\#\left( 22 \right) \end{aligned}$

The variance of this new model is

$\begin{aligned} \mathrm{Var} (\tilde{p}^{*})=L^{T}\mathrm{var} (\xi)L+L^{T}\mathrm{var} (\varepsilon)L=D^{*}\sigma_{\xi}^{2}+I\#\left( 23 \right) \end{aligned}$

The derivation of equation (23) is provided below. Since inverting this variance matrix and calculating the determinant of this matrix are simplified, high computational efficiency can be achieved. With the PSR method, the doubly iterative algorithm only applies once under the null model to deliver $\tilde{p}$, which is fit to the REML to scan the genome.

**Eigen decomposition of the kinship matrix for generalized linear mixed model**

For the *k*th marker, the linear mixed model for the pseudo response variable is

$$\begin{aligned} \tilde{p}=X\beta+Z_{k}\gamma_{k}+\xi+\varepsilon\#\left( 24 \right) \end{aligned}$$

where $Z_{k}$ is the genotype indicator variable for marker *k* and $\gamma_{k}$ is the marker effect for $k=1,\cdots,m$ and *m* is the total number of markers. The marker effect is now treated as a fixed effect. The random polygenic effect is $\xi=Z\gamma$, where $Z$ is an $n\times m$ matrix of marker genotype indicators and $\gamma$ is an $m\times1$ effects of genome-wide markers. The expectation of $\tilde{p}$ is $E(\tilde{p})=X\beta+Z_{k}\gamma_{k}$ and the variance is $var (\tilde{p})=V=K\sigma_{\xi}^{2}+R$. Under the null model, i.e., $\tilde{p}=X\beta+\xi+\varepsilon$, we perform the doubly iterative algorithm to generate the pseudo response variable $\tilde{p}$ (an $n\times1$ vector). Let us define $R^{-\frac{1}{2}}$ as the Cholesky decomposition of $R^{-1}$, i.e., $(R^{-\frac{1}{2}})^{T}R^{-\frac{1}{2}}=R^{-1}$. Define a new kinship matrix $K^{*}=(R^{-\frac{1}{2}})^{T}KR^{-\frac{1}{2}}$ and decompose $K^{*}$ so that $K^{*}=U^{*}D^{*}U^{*T}$. Now, let us define $L=R^{-\frac{1}{2}}U^{*}$ and pre-multiply all terms in (25) by $L^{T}$, we get

$\begin{aligned} \tilde{p}^{*}=L^{T}\tilde{p}=L^{T}X\beta+L^{T}Z_{k}\gamma_{k}+L^{T}\xi+L^{T}\varepsilon\#\left( 25 \right) \end{aligned}$

The variance of this new model is

$$\begin{aligned} \begin{aligned} \text{var} (L^{T}\tilde{p})=&L^{T}\text{var} (\xi)L+L^{T}\text{var} (\varepsilon)L \\ =&L^{T}KL\sigma_{\xi}^{2}+L^{T}RL \\ =&U^{*T}(R^{-\frac{1}{2}})^{T}KR^{-\frac{1}{2}}U^{*}\sigma_{\xi}^{2}+U^{*T}(R^{-\frac{1}{2}})^{T}RR^{-\frac{1}{2}}U^{*} \\ =&U^{*T}K^{*}U^{*}\sigma_{\xi}^{2}+U^{*T}(R^{-\frac{1}{2}})^{T}(R^{\frac{1}{2}})^{T}R^{\frac{1}{2}}R^{-\frac{1}{2}}U^{*} \\ =&U^{*T}U^{*}D^{*}U^{*T}U^{*}\sigma_{\xi}^{2}+U^{*T}U^{*} \\ =&D^{*}\sigma_{\xi}^{2}+I \end{aligned}\#\#(26) \\ \# \end{aligned}$$

Therefore, we have

$\begin{aligned} \text{var} (\tilde{p}^{*})=D^{*}\sigma_{\xi}^{2}+I\#\left( 27 \right) \end{aligned}$

which is diagonal and thus inverting this matrix and calculating the determinant of this matrix is straightforward.

**S1.7.3 Population parameters previously determined (P3D)**

With the P3D method, the doubly iterative algorithm only applies once under the null model to deliver $\hat{\sigma}_{\xi}^{2}$, which is treated as a known constant variance to scan the entire genome one marker at a time. The polygenic variance is a population parameter treated as a constant across all loci [13], but we still utilize the GLMM program to scan the genome.

**S1.8 QTL variance and heritability**

Recall that $\mathrm{var} (\tilde{p})=V=K\sigma_{\xi}^{2}+R$ and $P=V^{-1}-V^{-1}X\left( X^{T}V^{-1}X \right)X^{T}V^{-1}$. The estimated marker effect is $\hat{\gamma}_{k}=(Z^{T}PZ)^{-1}Z^{T}P\tilde{p}$ with a variance of $\mathrm{var} (\hat{\gamma}_{k})=(Z^{T}PZ)^{-1}$. We define the mean square of marker *k* (a putative QTL) by $MS_{G}={\hat{\gamma}_{k}^{T}Z^{T}PZ\gamma_{k}}/q$, where $q=1$. The mean squares of the residual errors is $MS_{E}={(\tilde{p}-Z\hat{\gamma}_{k})^{T}P\left( \tilde{p}-Z\hat{\gamma}_{k} \right)}/{(n-p-q})$, where $p$ is the number of columns of matrix $X$. Define the variance of the QTL by

$\begin{aligned} V_{QTL}={\gamma_{k}^{T}Z^{T}PZ\gamma_{k}}/{(n-p})\#\left( 28 \right) \end{aligned}$

The expectation of the QTL mean squares is $E\left( MS_{G} \right)={(n-p)\sigma_{QTL}^{2}}/q+1$. The expectation of the residual mean squares is $E\left( MS_{E} \right)=1=V_{E}$. Therefore, the estimated residual variance is $\hat{V}_{E}=MS_{E}$, although the expectation is 1. The estimated QTL variance is $\hat{V}_{QTL}={q\left( MS_{G}-MS_{E} \right)}/{(n-p)}$. The corresponding estimated QTL heritability is

$\begin{aligned} \hat{h}_{QTL}^{2}=\frac{\hat{V}_{QTL}}{\hat{V}_{QTL}+\hat{V}_{E}}=\frac{{q\left( MS_{G}-MS_{E} \right)}/\left( n-p \right)}{{q\left( MS_{G}-MS_{E} \right)}/\left( n-p \right)+MS_{E}}=\frac{MS_{G}-MS_{E}}{MS_{G}+{\left( n-p-q \right)MS_{E}}/q}\#\left( 29 \right) \end{aligned}$

The QTL heritability defined here is on the linear scale, not on the scale of observed trait. The derivation of the QTL heritability is provided below.

**S1.8.1 Variance Contributed by a QTL and the QTL Heritability**

Recall that the linear mixed model for the pseudo response variable is

$\begin{aligned} \tilde{p}=X\beta+Z_{k}\gamma_{k}+\xi+\varepsilon\#\left( 30 \right) \end{aligned}$

where $Z_{k}$ and $\gamma_{k}$ are the genotype indicator and the effect of marker *k*, for $k=1,\cdots,m$. Since we are focusing on the *k*th marker, we simply remove the subscript and redefine the model by

$\begin{aligned} \tilde{p}=X\beta+Z\gamma+\xi+\varepsilon\#\left( 31 \right) \end{aligned}$

The expectation and variance of $\tilde{p}$ are $E(\tilde{p})=X\beta+Z\gamma$ and

$\begin{aligned} \text{var} (\tilde{p})=V=K\sigma_{\xi}^{2}+R\#\left( 32 \right) \end{aligned}$

respectively. Let us define

$$\begin{aligned} P=V^{-1}-V^{-1}X(X^{T}V^{-1}X)^{-1}X^{T}V^{-1}\#\left( 33 \right) \end{aligned}$$

Define the variance for marker *k* (QTL variance) by

$\begin{aligned} V_{QTL}=\frac{1}{n-p}\gamma^{T}Z^{T}PZ\gamma\#\left( 34 \right) \end{aligned}$

where $n$ is the sample size and $p$ is the number of columns of matrix *X*. The mean squares of the marker and the residual are

$\begin{aligned} MS_{G}=\frac{1}{q}\hat{\gamma}^{T}Z^{T}PZ\gamma\#\left( 35 \right) \end{aligned}$

and

$\begin{aligned} MS_{E}=\frac{1}{n-p-q}(y-Z\hat{\gamma})^{T}P\left( y-Z\gamma\right)\#\left( 36 \right) \end{aligned}$

respectively, where $q=1$ is the number of effects for the marker. If dominance effect is included, $q=2$. Theoretically, $q$ may be greater than 2 for a population generated from multiple parents. The expectation of the QTL mean square is

$$\begin{aligned} \begin{aligned} \text{E}\left( MS_{G} \right)=&\frac{\text{1}}{\text{q}}\text{E}\left( \hat{\gamma}^{T}Z^{T}PZ\gamma\right) \\ =&\frac{1}{q}\left\{ \text{E}\left( \hat{\gamma}^{T} \right)Z^{T}PZ\text{E}\left( \gamma\right)+\text{tr}\left[ \text{var} (\hat{\gamma})Z^{T}PZ \right] \right\} \\ =&\frac{1}{q}\left\{ \gamma^{T}Z^{T}PZ\gamma+\text{tr}\left[ (Z^{T}PZ)^{-1}Z^{T}PZ \right] \right\} \\ =&\left\{ \frac{n-p}{q\left( n-p \right)}\gamma^{T}Z^{T}PZ\gamma+\frac{1}{q}\text{tr}\left( I \right) \right\} \\ =&\left\{ \frac{n-p}{q}V_{QTL}+\frac{1}{q}\text{tr}\left( I \right) \right\} \\ =&\left\{ \frac{n-p}{q}V_{QTL}+\frac{q}{q} \right\} \\ =&\frac{n-p}{q}V_{QTL}+1 \end{aligned}\#\#(37) \\ \# \end{aligned}$$

The expectation of the residual mean square is

$$\begin{aligned} \begin{aligned} \text{E}\left( MS_{E} \right)=&\frac{1}{n-p-q}\text{E}\left[ (y-Z\hat{\gamma})^{T}P(y-Z\gamma) \right] \\ =&\frac{1}{n-p-q}\left\{ \text{E}(y-Z\hat{\gamma})^{T}P\text{E}(y-Z\gamma)+\text{tr}\left[ \text{var} (y-Z\gamma)P \right] \right\} \\ =&\frac{1}{n-p-q}\left[ \text{E}(y-Z\hat{\gamma})^{T}P\text{E}(y-Z\gamma)+\text{tr}\left[ \text{var} (y-Z\gamma)P \right] \right] \\ =&\frac{1}{n-p-q}\left[ \beta^{T}X^{T}PX\beta+\text{tr}\left( VP \right) \right] \\ =&\frac{1}{n-p-q}\text{tr}\left( VP \right) \\ =&\frac{n-p-q}{n-p-q} \\ =&1 \\ =&V_{E} \end{aligned}\#\#(38) \end{aligned}$$

This is due to the fact that

$$\begin{aligned} \begin{aligned} \text{t}\text{r}\left( PV \right)=&\text{tr}\left\{ V\left[ V^{-1}-V^{-1}X(X^{T}V^{-1}X)^{-1}X^{T}V^{-1} \right] \right\} \\ =&\text{tr}\left[ VV^{-1}-X(X^{T}V^{-1}X)^{-1}X^{T}V^{-1} \right] \\ =&\text{tr}\left( VV^{-1} \right)-\text{tr}\left[ X(X^{T}V^{-1}X)^{-1}X^{T}V^{-1} \right] \\ =&\text{tr}\left( I_{n} \right)-\text{tr}\left[ (X^{T}V^{-1}X)^{-1}X^{T}V^{-1}X \right] \\ =&\text{tr}\left( I_{n} \right)-\text{tr}\left[ X^{T}V^{-1}X(X^{T}V^{-1}X)^{-1} \right] \\ =&n-p-q \end{aligned}\#\#(39) \end{aligned}$$

Therefore, the estimated residual variance is

$\begin{aligned} \hat{V}_{E}=MS_{E}\#\left( 40 \right) \end{aligned}$

The estimated QTL variance is

$\begin{aligned} \hat{V}_{QTL}=\frac{q}{n-p}\left( MSG-MSE \right)\#\left( 41 \right) \end{aligned}$

The corresponding estimated QTL heritability is

$\begin{aligned} \hat{h}_{QTL}^{2}=\frac{\hat{V}_{QTL}}{\hat{V}_{QTL}+V_{E}}=\frac{{q\left( MS_{G}-MS_{E} \right)}/\left( n-p \right)}{{q\left( MS_{G}-MS_{E} \right)}/\left( n-p \right)+MS_{E}}=\frac{MS_{G}-MS_{E}}{MS_{G}+{\left( n-p-q \right)MS_{E}}/q}\#\left( 42 \right) \end{aligned}$

**S1.9 Evaluation of bias**

The pseudo response method for GLMM is biased in estimated parameters for binary response variable [14,15]. To show the level of bias and the effect of the bias on the GWAS result, we performed a simulation study. We took the IMF2 hybrid rice genotype data (*n* = 278 plants and *m* = 1619 markers) to calculate the kinship matrix. We added a true polygenic variance with a value of 1.0 ($\sigma_{\xi}^{2}=1.0$) and a residual variance with a true value of 1.0 ($\sigma^{2}=1.0$). The polygenic effects and the residual errors were simulated from their respective variances (normal distributions) to form a quantitative trait. On the simulated quantitative trait, we added a marker (Bin728 of the rice data) with a known effect of $\gamma_{728}=0.70$ or $\gamma_{728}=1.0$, respectively (two cases). The former explained about 10% of the variance for the simulated trait ($h_{QTL}^{2}=0.10$) and the latter explained about 18% of the variance for the simulated trait ($h_{QTL}^{2}=0.18$). Finally, we defined a binary outcome by setting the binary trait value equal to 0 if the quantitative trait value was below 0 and 1 if the quantitative trait value was above 0. The GWAS was performed on the simulated binary trait. The simulation of each case (either $h_{QTL}^{2}=0.10$ or $h_{QTL}^{2}=0.18$) was replicated 100 times. We compared the estimated parameters and the test statistics of four methods, (1) The pseudo response variable (PSR) approach implemented with PROC GLIMMIX METHOD = RSPL in SAS; (2) The Laplace (LAPLACE) method to approximate the marginal likelihood function implemented with PROC GLIMMIX METHOD = LAPLACE in SAS; (3) The Bayesian generalized linear mixed model (BGLIMM) implemented with PROC BGLIMM in SAS under the default prior with 1000 iterations of MCMC after deleting 500 iterations as burn in and thinning the chain by keeping one observation in every 10 iterations. (4) The Bayesian method implemented with the BGLR package in R [16]. The number of burn-ins, the number of MCMC iterations and the thinning rate of the BGLR were the same as the BGLIMM method. The BGLR package treated the underlying liability as missing values, which were then sampled from the conditional posterior distributions, which were truncated normal distributions [17]. Technically, results of the Bayesian analysis should not be compared with the results of the likelihood method in terms of significance test. Bayesian analysis produced posterior distribution of the parameters. We used a Bayesian “t-test” to compare it with the t-test of the frequentist approach, where the Bayesian t-test is the posterior mean of the QTL effect divided by the posterior standard deviation of the QTL effect. We provided the SAS code used to simulate and analyze the data in **Supplementary Code S2**.

**S1.10 Power study**

To study the power and type 1 error, we conducted a separate simulation experiment comparing GLMM and PSR with GMMAT [18] for binary traits. We also compared GLMM and PSR with POLMM [19] for ordinal traits. We used the kinship matrix calculated from the 1619 bins of the hybrid rice population. We added a polygenic variance of $\sigma_{\xi}^{2}=1$ and a residual variance of $\sigma^{2}=1$ to simulate the polygenic effects and the residual errors. We used Bin728 (locus) as the simulated QTL and added an effect to this locus (additive effect). The effect ranged from 0 to 0.75 incremented by 0.15. The QTL heritability corresponding to the effects are shown in **Supplementary Table 1**.

**Supplementary Table 1** Simulated QTL effects and the corresponding QTL heritability for power studies

| QTL effect ($\gamma_{728}$) | Heritability ($h_{QTL}^{2}$) |
| --- | --- |
| 0.00 | 0.0000 |
| 0.15 | 0.0050 |
| 0.30 | 0.0198 |
| 0.45 | 0.0436 |
| 0.60 | 0.0749 |
| 0.75 | 0.1123 |

The simulated quantitative trait was considered as the underlying liability and converted to a binary phenotype with 1 if the liability is greater than 0 and 0 if the liability is less than 0. The ordinal trait was also formed from the simulated quantitative trait by dividing the liability into three equal area clusters, as shown below,

$\text{Ordinal}=\left\{ \begin{matrix} 0 \\ 1 \\ 2 \end{matrix} \right.\begin{matrix} \\ \\ \end{matrix}\begin{matrix} \text{if} \\ \text{if} \\ \text{if} \end{matrix}\begin{matrix} \\ \\ \end{matrix}\begin{matrix} \text{liability}<-0.439913 \\ -0.439913<\text{liability}<0.439913 \\ 0.439913<\text{liability} \end{matrix}$

The proportion of the variance of the underlying quantitative trait explained by the simulated marker ranged from 0 to 0.11 (see **Supplementary Table 1**). The QTL variance was calculated by

$\sigma_{QTL}^{2}=\mathrm{var} (Z_{728})\gamma_{728}^{2}=0.45\times\gamma_{728}^{2}$

where $\gamma_{728}^{2}$ is the QTL effect (**Supplementary Table 1**) and $\mathrm{var} (Z_{728})\approx0.45$ is the genotype indicator of Bin728. Theoretically, this variance is 0.50 but the realized variance was 0.45 instead. The kinship matrix *K* has been normalized so that the trace of *K* equals the sample size. Therefore, the underlying liability variance was

$\mathrm{var} (\text{liability})=\mathrm{var} (Z_{728}\gamma_{728})+\mathrm{var} (\xi)+\mathrm{var} (\varepsilon)=0.45\gamma_{728}^{2}+\sigma_{\xi}^{2}+\sigma^{2}=0.45\gamma_{728}^{2}+1+1$

The QTL heritability was defined as

$h_{QTL}^{2}=\frac{0.45\gamma_{728}^{2}}{0.45\gamma_{728}^{2}+1+1}$

We also added a locus (Bin1619) to the model with no QTL effect to monitor the type 1 error. The null marker (Bin1619) was not in linkage disequilibrium (LD) with the true marker (Bin728) because the realized correlation between Bin728 and Bin1619 was -0.02 with a p-value of near 0.99. The actual liability model used in the simulation was

$$Liability=\mu+Z_{728}\gamma_{728}+Z_{1619}\gamma_{1619}+\xi+\varepsilon$$

where $\gamma_{1619}=0$ and $\gamma_{728}$ took various values listed in **Supplementary Table 1**. Under each level of the simulated QTL effect, a receiver operating characteristic (ROC) curve was drawn from 1000 replicated simulation results for each method. We ranked the Wald test statistics for Bin1619 (zero effect) in descending order for the 1000 replicates. When the type 1 error is zero, the power is also zero. When the largest Wald test of the 1000 replicates of the null marker was set as the critical value of significance, the proportion of the 1000 replicates of the true QTL (Bin728) that surpassed this critical value was the power corresponding to the type 1 error of 0.1%. When the second highest Wald test from the null marker was set as the critical value, the proportion of the replicates of the true QTL (Bin728) that surpassed the critical value was the power corresponding to the type 1 error of 0.2%. This process continued until the lowest Wald test of the null marker was set as the critical value, the proportion of the replicates of the true QTL surpassed this critical value was the power corresponding to the type 1 error of 100%. The area under the curve (AUC) indicates the power of a method, the larger the area, the higher the power. The R code performing the simulation experiments for the power analysis is given in **Supplementary Code S3.**

**Supplementary Note S2: Converting kinship matrix model into general-purpose linear mixed model**

We separated the null model from the main package “PSR-GLMM/R” and made it a general package for generalized linear mixed model analysis. Two additional features were added to the general package: (1) The fixed effects included the intercept and user defined additional fixed effects (if they exist), e.g., location, year, age and so on. (2) The covariance structure defined by the kinship matrix is special for GWAS and QTL mapping. The general purpose GLMM does not have to include a covariance structure captured by the kinship matrix. We used a spectral decomposition for the kinship matrix, $K=UDU^{T}=UD^{\frac{1}{2}}D^{\frac{1}{2}}U^{T}=ZZ^{T}$, where $Z=UD^{\frac{1}{2}}$ and $D^{\frac{1}{2}}=\sqrt{D}$. We then defined the linear predictor by $\eta=X\beta+Z\gamma$ instead of $\eta=X\beta+\xi$, where $\text{var} (\gamma)=I\sigma_{\gamma}^{2}$. The pseudo response variable is described as$\tilde{p}=X\beta+Z\gamma+\varepsilon$. The variance of the pseudo response is

$\begin{aligned} \text{var} (\tilde{p})=ZZ^{T}\sigma_{\gamma}^{2}+R=UD^{\frac{1}{2}}D^{\frac{1}{2}}U^{T}\sigma_{\gamma}^{2}+R=K\sigma_{\gamma}^{2}+R\#\left( 1 \right) \end{aligned}$

Dealing with the design matrix *Z* for random effects is much more convenient than the kinship matrix. If users want to use PSR-GLMM/R for a general-purpose analysis, this null model function with the *Z* design matrix should be called. In the main package, users can call the general purpose GLMM for analyzing non-normal response variables. Users are required to provide both the *X* matrix and the *Z* matrix. In addition, The BLUP for the random effects in the general-purpose model are obtained differently from the kinship matrix approach, as shown below,

$\begin{aligned} \hat{\gamma}=\sigma_{\gamma}^{2}Z^{T}(ZZ^{T}\sigma_{\gamma}^{2}+R)^{-1}\left( \tilde{p}-X\hat{\beta} \right)\#\left( 2 \right) \end{aligned}$

Note that the original version of the BLUP for the polygene was

$\begin{aligned} \hat{\xi}=\sigma_{\xi}^{2}K(K\sigma_{\xi}^{2}+R)^{-1}\left( \tilde{p}-X\hat{\beta} \right)\#\left( 3 \right) \end{aligned}$

The difference between the two is clearly demonstrated between equations (2) and (3). Since $\hat{\xi}=Z\hat{\gamma}$, pre-multiplying equation (2) by *Z* leads to

$\begin{aligned} Z\hat{\gamma}=\sigma_{\gamma}^{2}ZZ^{T}(ZZ^{T}\sigma_{\gamma}^{2}+R)^{-1}(\tilde{p}-X\hat{\beta})=\sigma_{\gamma}^{2}K(K\sigma_{\gamma}^{2}+R)^{-1}\left( \tilde{p}-X\hat{\beta} \right)\#\left( 4 \right) \end{aligned}$

where $\sigma_{\gamma}^{2}=\sigma_{\xi}^{2}$ represent the same variance parameter.

**Supplementary Note S3: Theoretical computational complexity**

Let $n$ be the number of individuals, $m$ be the number of markers, and $C$ be the number of ordered categories. With the cumulative probit link function, the working response dimension is $N=n(C-1)$. The overall computational characteristics are summarized as follows.

| Method | Core principle | Time complexity | Memory | Scalability |
| --- | --- | --- | --- | --- |
| GLMM | Per-marker re-fitting with variance-component estimation | $\mathcal{O(}t_{\text{alt}}\text{ }m\text{ }N^{3})$ | $\Theta(N^{2})$ | Not feasible at biobank scale |
| P3D | Fit null model once and reuse variance components | One-off $\mathcal{O(}t_{\text{null}}\text{ }N^{3})$, then per-marker $\mathcal{O(}Nq)$ | $\Theta(N^{2})$ | Linear per-marker; feasible for 100 K × 5 M |
| PSR | Spectral diagonalization (project–solve–reconstruct) | One-off $\mathcal{O(}N^{3})$or truncated $\tilde{\mathcal{O}}(N^{2}r+r^{3})$; per-marker $\mathcal{O(}N)$ | $\Theta(Nr)$ | Most efficient; highly parallelizable |

Here, $t_{\text{null}}$ and $t_{\text{alt}}$denote the number of iterations for the null and alternative models, respectively, $q=C$ is the number of fixed coefficients (thresholds + marker effect), and $r$ is the truncated spectral rank of the genomic relationship matrix (GRM).

- GLMM: Re-estimates variance components per marker, yielding cubic cost $\mathcal{O}\left( N^{3} \right)$and quadratic memory, making it impractical for $n>{10}^{4}$.
- P3D: The cubic operation occurs only once during null-model fitting; subsequent marker tests scale linearly with $n$.
- PSR: After a single spectral decomposition, the transformed covariance is diagonal, and each marker test becomes a lightweight weighted regression, giving near-linear scaling in $n$ with a very small constant factor.

We now translate the complexity into empirical scaling and projection to biobank-scale. The empirical results from both the simulation study and the four real datasets align closely with the theoretical complexity analysis. In the simulated rice hybrid population $(n\approx278,\text{ }m=1619)$, PSR achieves 100–300× speed-up over the exact GLMM and 10–30× over P3D, while producing nearly identical test statistics and effect-size estimates. In the real-data applications (*Arabidopsis*, pigs, and dogs; $(n=80\text{–}200,\text{ }m\approx50\text{K–}216\text{K})$), PSR again delivers one to two orders of magnitude improvement over GLMM and substantial gains over P3D, with highly concordant Manhattan plots and detected QTL across methods. These results confirm that the computational advantages of PSR do not compromise inference accuracy. Extrapolating these results to biobank-scale datasets ($n\sim{10}^{5},m\sim{10}^{6}$) yields a clear scalability profile:

- GLMM requires repeated variance-component estimation and dense matrix factorization, resulting in week-long runtimes and terabyte-level memory usage—thus infeasible at biobank scale.
- P3D is partially scalable but remains constrained by its single $\mathcal{O(}N^{3})$ null-model fit, especially with multiple ordinal thresholds.
- PSR (proposed method) maintains near-linear scalability with respect to sample size during the genome-wide scanning stage. Its projected runtime at biobank scale is only a few hours. More importantly, the per-marker computations are embarrassingly parallel, allowing workload distribution at the chromosome level or across marker blocks. With a moderately sized computing cluster (e.g., tens to hundreds of CPU cores), the analysis can be further accelerated.

These findings confirm that PSR provides a computationally practical framework for biobank-scale GWAS of discrete and ordinal traits without sacrificing inferential reliability.

**References**

- 1. Hua JP, Xing YZ, Xu CG et al. Genetic dissection of an elite rice hybrid revealed that heterozygotes are not always advantageous for performance. *Genetics* 2002; 162: 1885–95.
  2. VanRaden PM. Efficient methods to compute genomic predictions. *J Dairy Sci* 2008; 91: 4414–23.
  3. Weng J, Gu S, Wan X et al. Isolation and initial characterization of GW5, a major QTL associated with rice grain width and weight. *Cell Res* 2008; 18: 1199–1209.
  4. Xu S. Mapping quantitative trait loci by controlling polygenic background effects. *Genetics* 2013; 195: 1209–22.
  5. Koopman BO. On distributions admitting a sufficient statistic. *Trans Am Math Soc* 1936; 39: 399–409.
  6. Wolfinger R, O'Connell M. Generalized linear mixed models: a pseudo-likelihood approach. *J Stat Comput Simul* 1993; 48: 233–43.
  7. Finney DJ. Probit analysis: a statistical treatment of the sigmoid response curve. Cambridge: Cambridge University Press; 1952.
  8. Patterson HD, Thompson R. Recovery of inter-block information when block sizes are unequal. *Biometrika* 1971; 58: 545–54.
  9. Liu C. Robit regression: a simple robust alternative to logistic and probit regression. In: Gelman A, Meng XL (eds). Applied Bayesian Modeling and Causal Inference from Incomplete-Data Perspectives. Chichester: Wiley; 2004: 227–38.
  10. Kang HM, Zaitlen NA, Wade CM et al. Efficient control of population structure in model organism association mapping. *Genetics* 2008; 178: 1709–23.
  11. Lippert C, Listgarten J, Liu Y et al. FaST linear mixed models for genome-wide association studies. *Nat Methods* 2011; 8: 833–35.
  12. Zhou X, Stephens M. Genome-wide efficient mixed-model analysis for association studies. *Nat Genet* 2012; 44: 821–24.
  13. Zhang Z, Ersoz E, Lai CQ et al. Mixed linear model approach adapted for genome-wide association studies. *Nat Genet* 2010; 42: 355–60.
  14. Breslow NE, Lin X. Bias correction in generalised linear mixed models with a single component of dispersion. *Biometrika* 1995; 82: 81–91.
  15. Lin X, Breslow NE. Bias correction in generalized linear mixed models with multiple components of dispersion. *J Am Stat Assoc* 1996; 91: 1007–16.
  16. Pérez P, de Los Campos G. Genome-wide regression and prediction with the BGLR statistical package. *Genetics* 2014; 198: 483–95.
  17. Albert JH, Chib S. Bayesian analysis of binary and polychotomous response data. *J Am Stat Assoc* 1993; 88: 669–79.
  18. Chen H. GMMAT: generalized linear mixed model association tests (R package, Version 1.5.0). 2025.
  19. Bi W, Zhou W, Dey R et al. Efficient mixed model approach for large-scale genome-wide association studies of ordinal categorical phenotypes. *Am J Hum Genet* 2021; 108: 825–39.
