# Supplementary figures and images for: Genome-wide association studies and QTL mapping for traits deviating from normal distribution
Source: Natl Sci Rev. 2026 Mar 23;13(10):nwag184. doi: 10.1093/nsr/nwag184 (PMC13218386; doi:10.1093/nsr/nwag184)

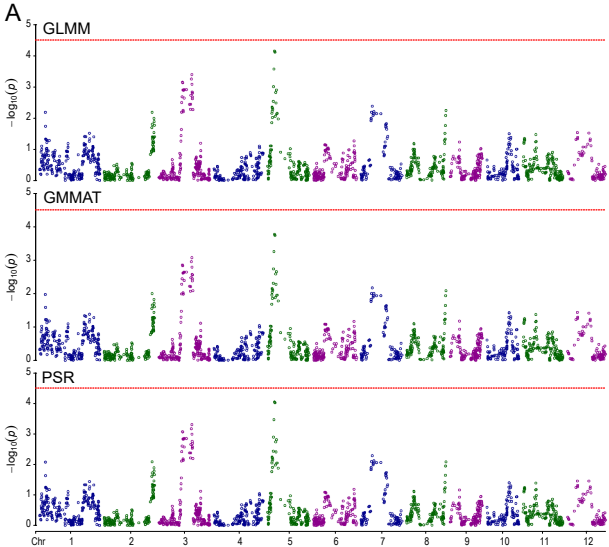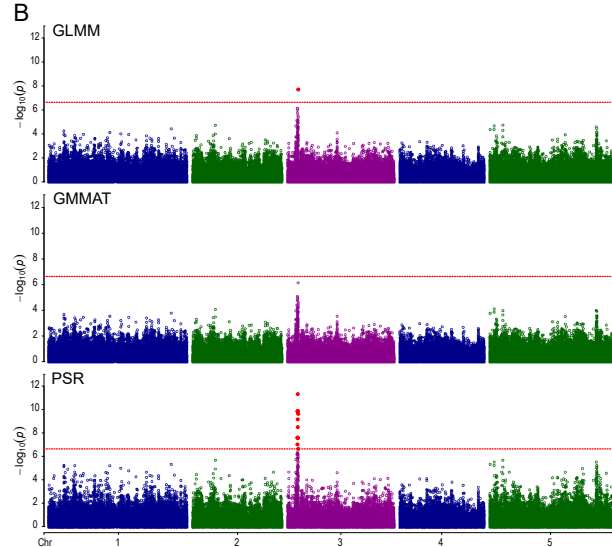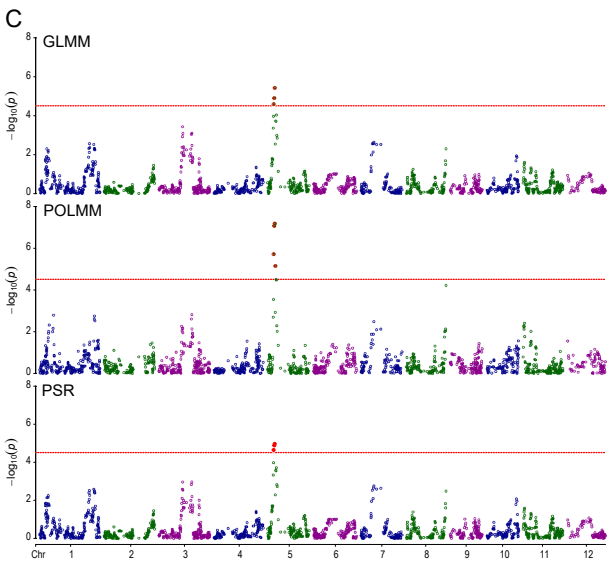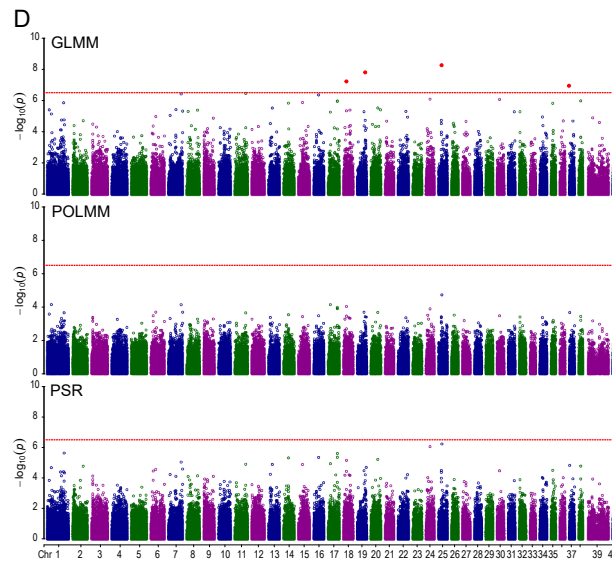

Supplement: nwag184_Supplemental_Files [file nwag184_supplemental_files.zip › Supplementary Figure S1.pdf]

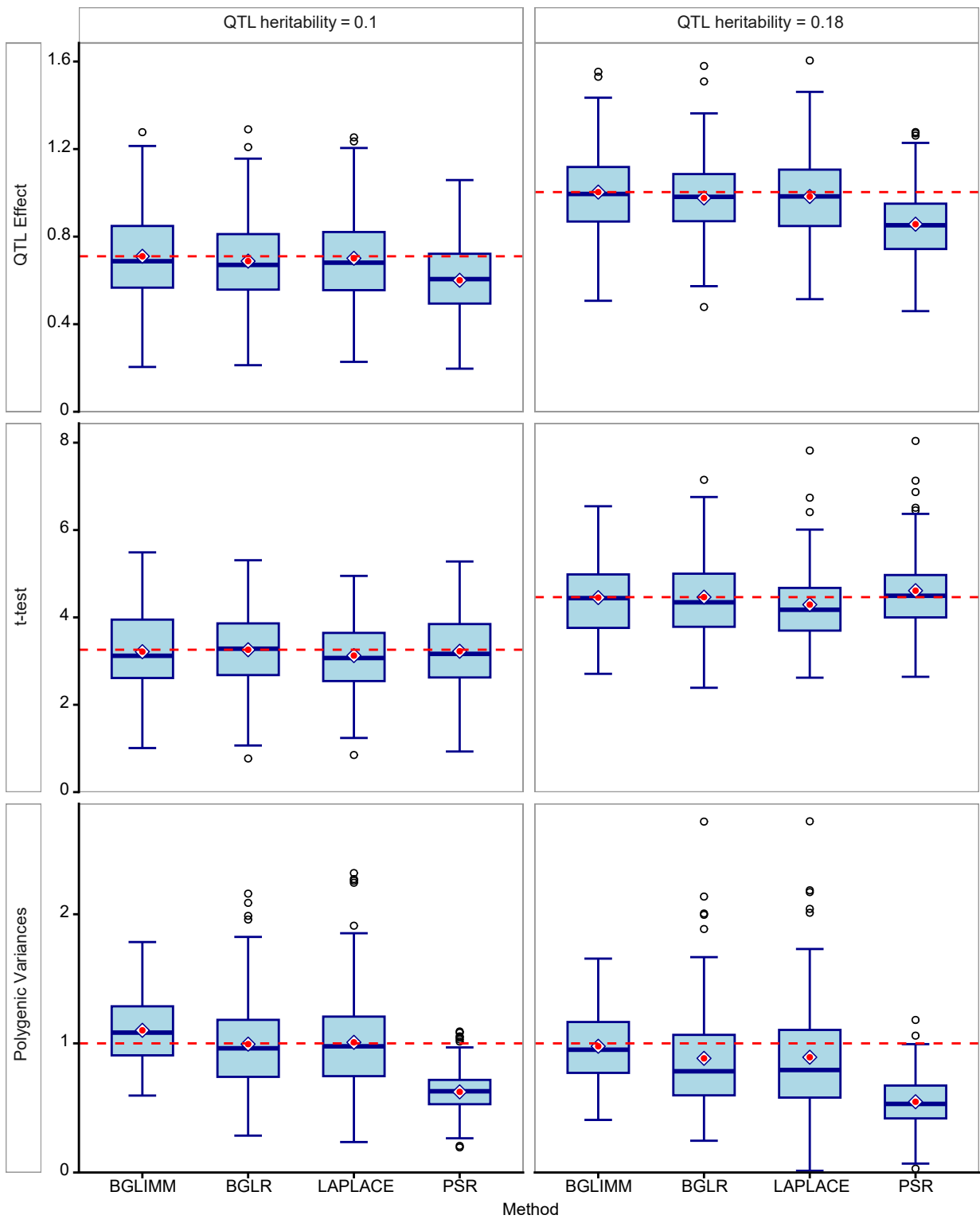

Supplement: nwag184_Supplemental_Files [file nwag184_supplemental_files.zip › Supplementary Figure S2.pdf]

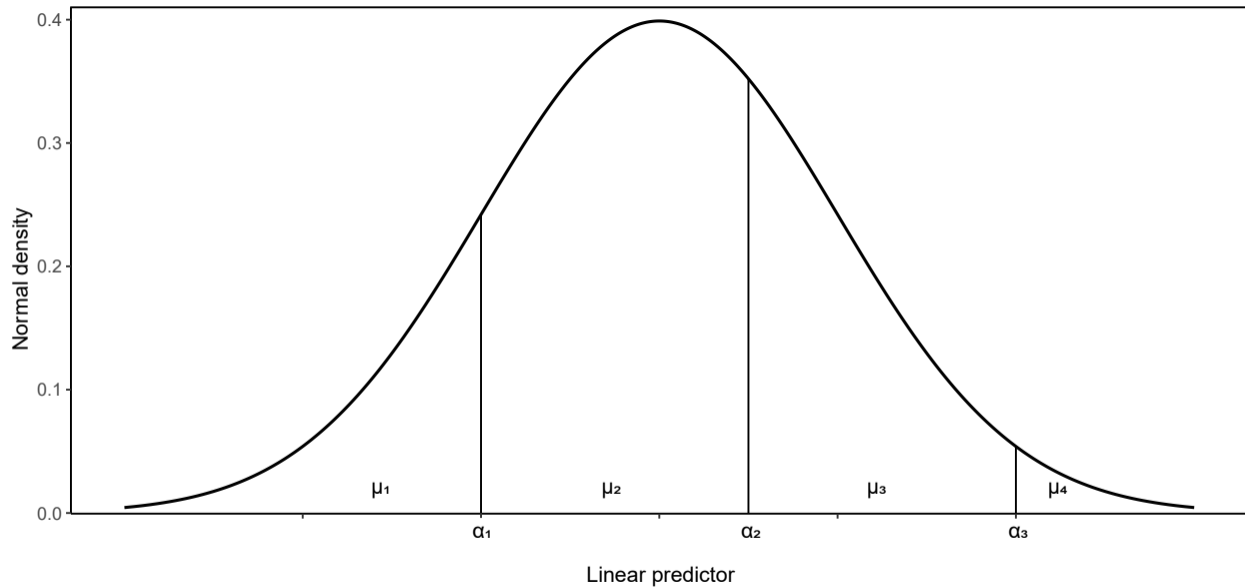

Supplement: nwag184_Supplemental_Files [file nwag184_supplemental_files.zip › Supplementary Figure S3.pdf]
